# Supplementary material for: Stereoselective [4+3]-Cycloaddition of 2-Amino-β-nitrostyrenes with Azaoxyallyl Cations to Access Functionalized 1,4-Benzodiazepin-3-ones
Source: Molecules. 2024 Mar 8;29(6):1221. doi: 10.3390/molecules29061221 (PMC10975549; doi:10.3390/molecules29061221)
Supplement: Supplementary file 1 [file molecules-29-01221-s001.zip › molecules-2906758-supplementary.pdf]

Supporting Information

Stereoselective [4+3]-Cycloaddition of 2-Amino-β-Nitrostyrenes with Azaoxyallyl Cations to Access Functionalized 1,4-Benzodiazepin-3-Ones

Yoseop Kim, and Sung-Gon Kim*

Department of Chemistry, College of Natural Science, Kyonggi University, 154-42, Gwanggyosan-ro,Yeongtong-gu, Suwon-si, Gyeonggi-do 16227, Republic of Korea

X-ray Crystallography Data of Compound 3a

Sample preparation: The single crystal **3a** was obtained by slow diffusion of *n*-hexane in to a solution of **3a** in EtOAc at room temperature.

Crystal measurement: A crystal of **3a** was picked up with paratone oil and mounted on a Bruker D8 Venture PHOTON III M14 diffractometer equipped with a graphite-monochromated Mo Kα (λ = 0.71073 Å) radiation source and a nitrogen cold stream (223 K). Data collection and integration Data collection and integration were performed with SMART APEX3 (Bruker, 2016) and SAINT (Bruker, 2016). The absorption correction was performed by a multi-scan method implemented in SADABS. The structure was solved by direct methods and refined by full-matrix least-squares on F^2 using SHELXTL. All the non-hydrogen atoms were refined anisotropically, and the hydrogen atoms were added to their geometrically ideal positions. Thermal ellipsoids are drawn at 30% probability level.

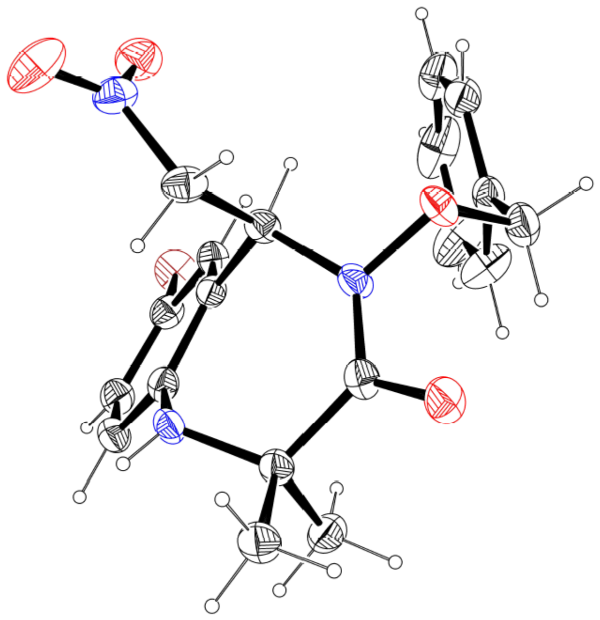

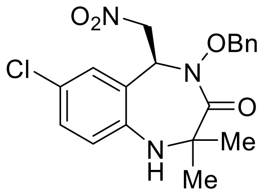

3a

Identification code	KGU_23_07_a
Empirical formula	C19 H20 Br N3 O4
Formula weight	434.29
Temperature	223(2) K
Wavelength	0.71073 Å
Crystal system	Orthorhombic

Space group	$P2_12_12_1$	
Unit cell dimensions	a = 9.3534(4) Å	α= 90°.
	b = 12.7616(6) Å	β= 90°.
	c = 16.4794(8) Å	γ = 90°.
Volume	1967.05(16) Å^3	
Z	4	
Density (calculated)	1.466 Mg/m^3	
Absorption coefficient	2.119 mm^{-1}	
F(000)	888	
Crystal size	0.118 x 0.090 x 0.085 mm^3	
Theta range for data collection	2.018 to 28.295°.	
Index ranges	-9<=h<=12, -17<=k<=17, -21<=l<=21	
Reflections collected	47516	
Independent reflections	4882 [R(int) = 0.1107]	
Completeness to theta = 25.242°	100.0 %	
Absorption correction	Semi-empirical from equivalents	
Max. and min. transmission	0.7323 and 0.6937	
Refinement method	Full-matrix least-squares on F^2	
Data / restraints / parameters	4882 / 0 / 250	
Goodness-of-fit on F^2	0.876	
Final R indices [I>2sigma(I)]	R1 = 0.0417, wR2 = 0.1097	
R indices (all data)	R1 = 0.1027, wR2 = 0.1522	
Absolute structure parameter	0.009(6)	
Extinction coefficient	n/a	
Largest diff. peak and hole	0.279 and -0.547 e.Å^{-3}	

^{1}H NMR (400 MHz) in $CDCl_3$

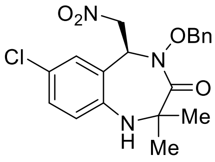

3a

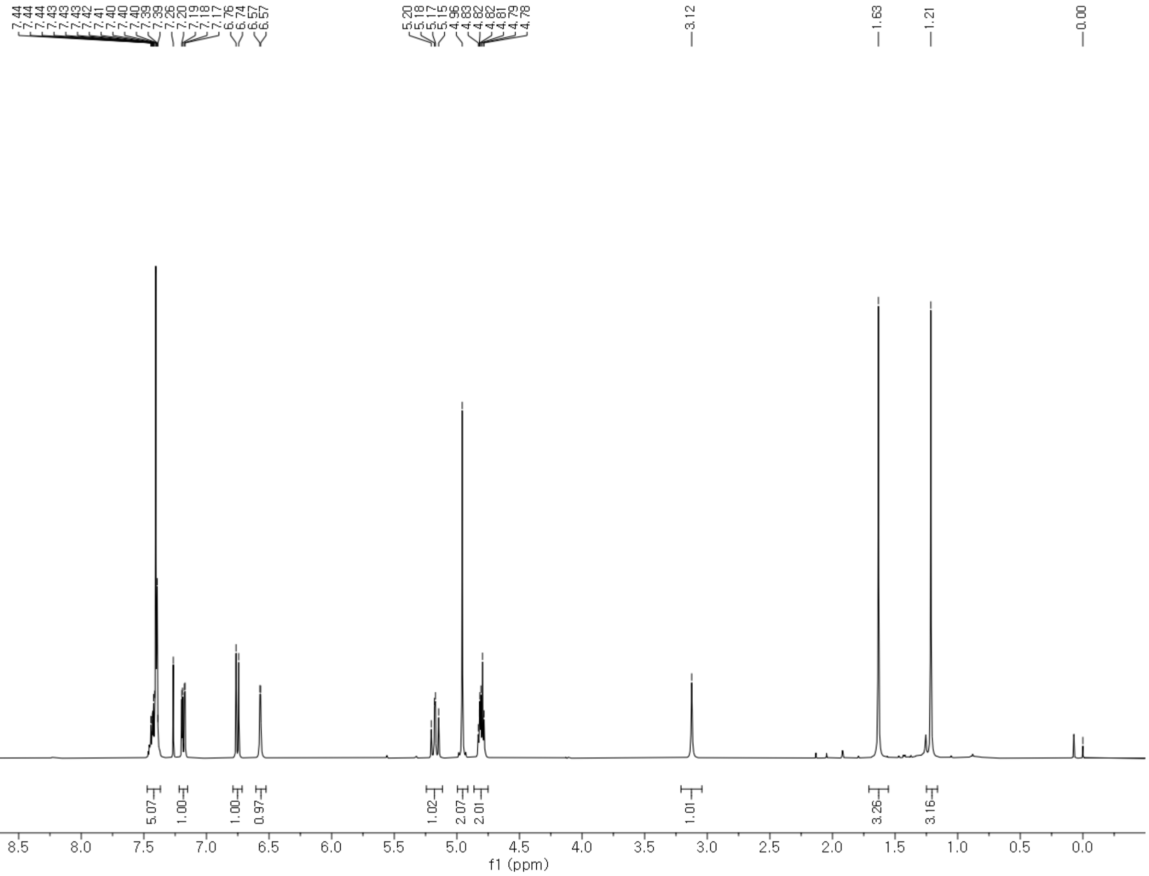

^{13}C NMR (101 MHz) in $CDCl_3$

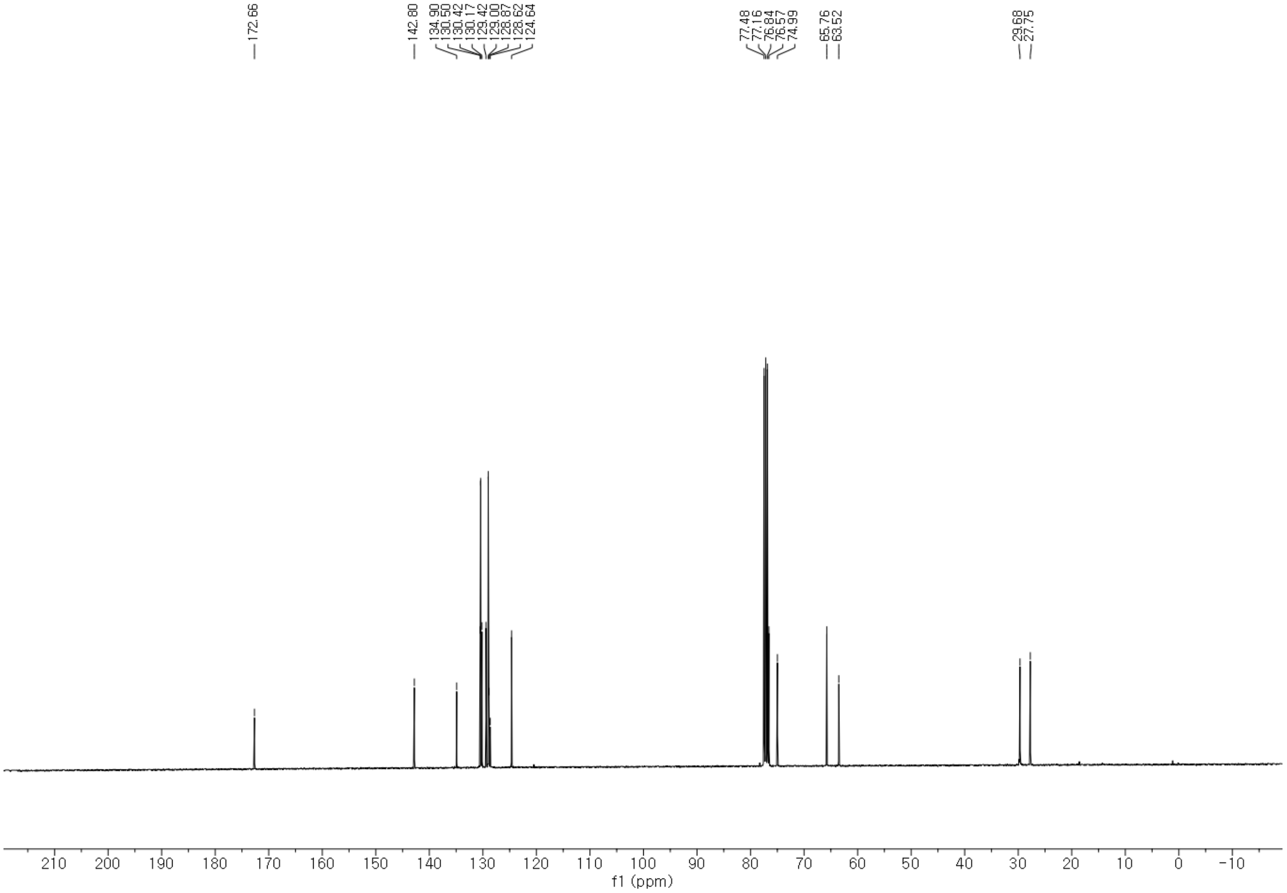

^{1}H NMR (400 MHz) in $CDCl_3$

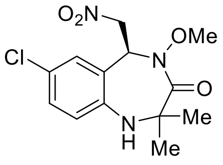

3b

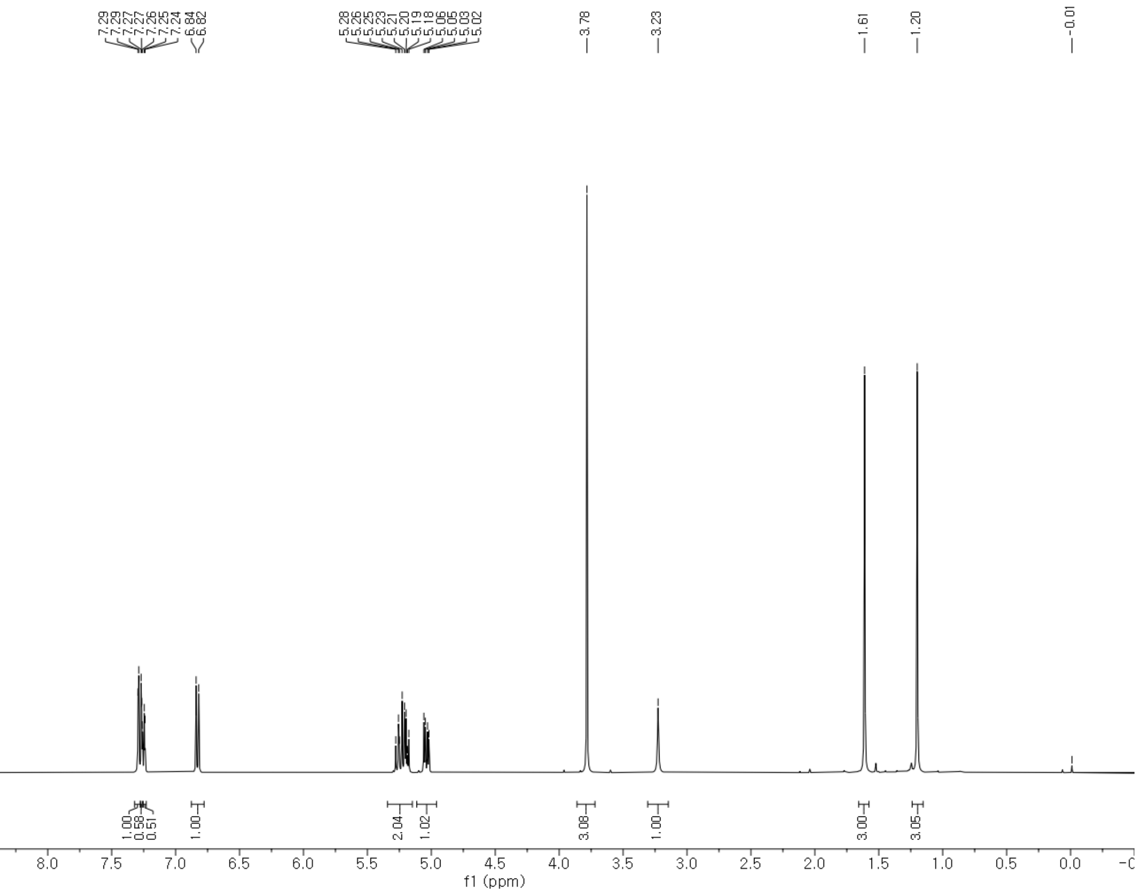

^{13}C NMR (101 MHz) in $CDCl_3$

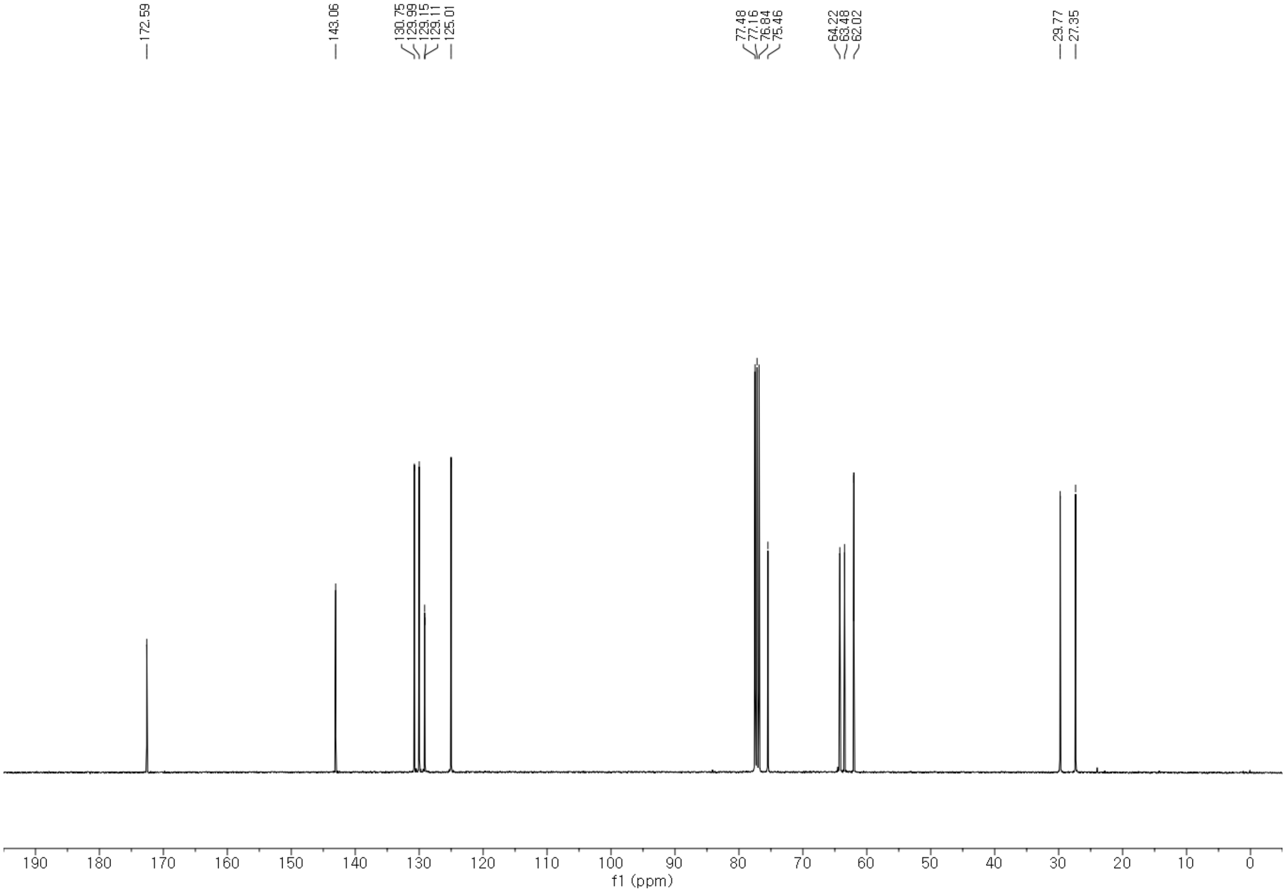

^{1}H NMR (400 MHz) in $CDCl_3$

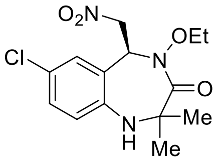

3c

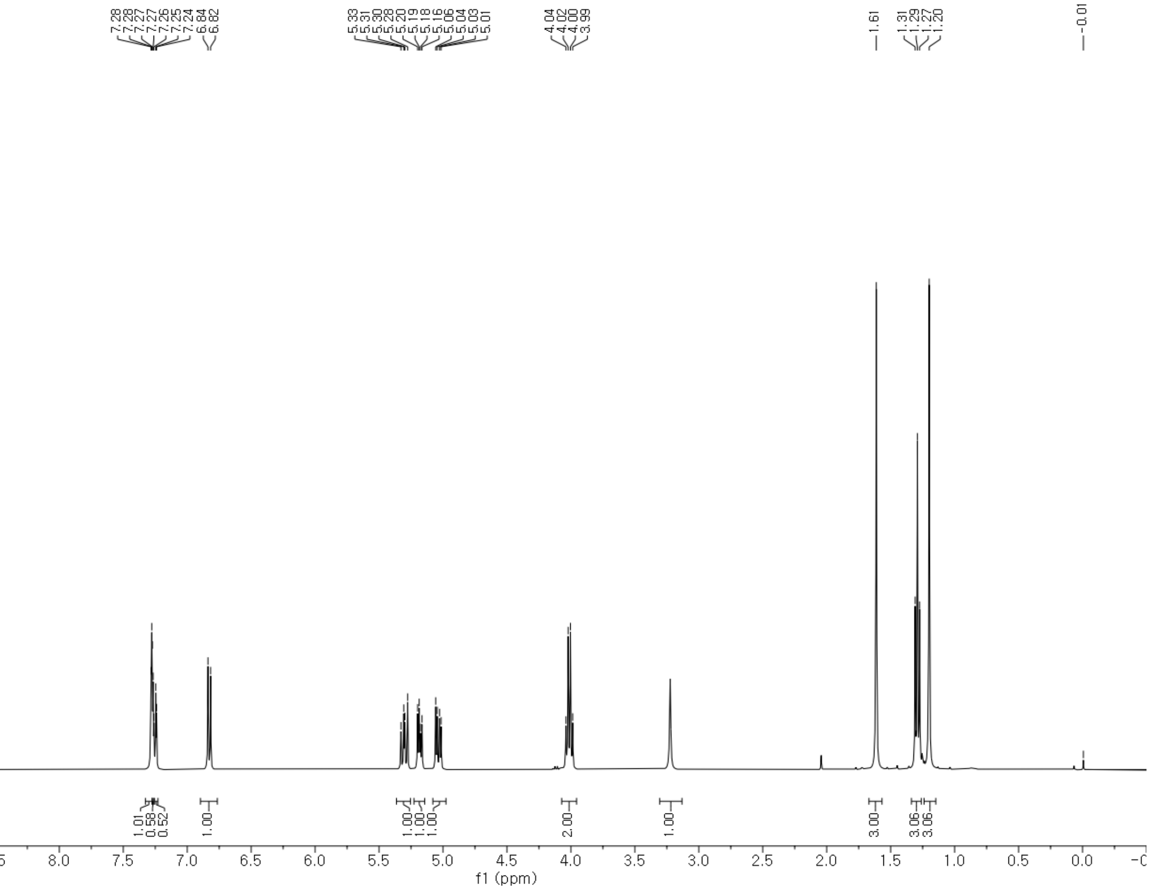

^{13}C NMR (101 MHz) in $CDCl_3$

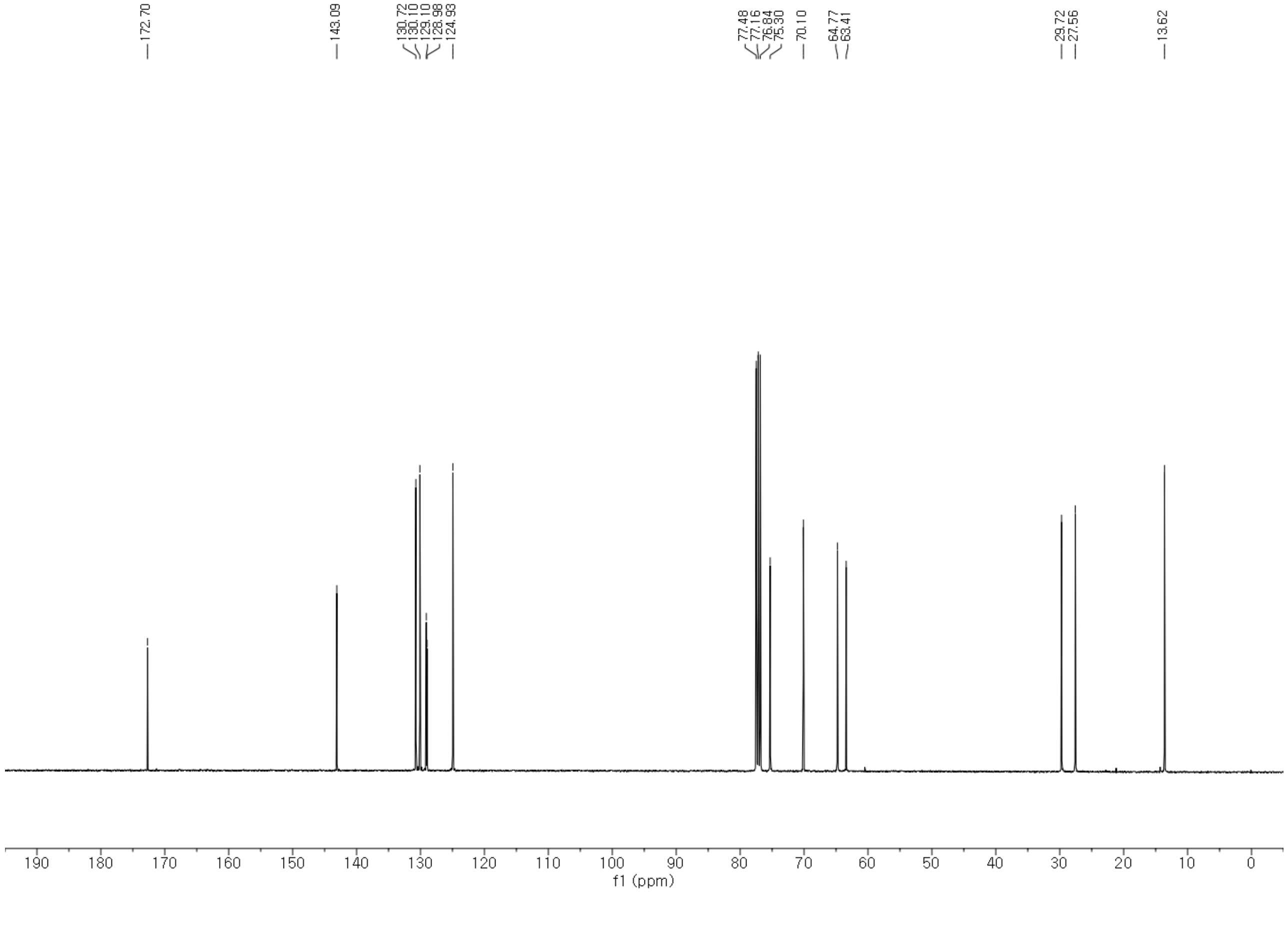

^{1}H NMR (400 MHz) in $CDCl_3$

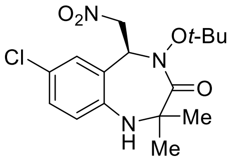

3d

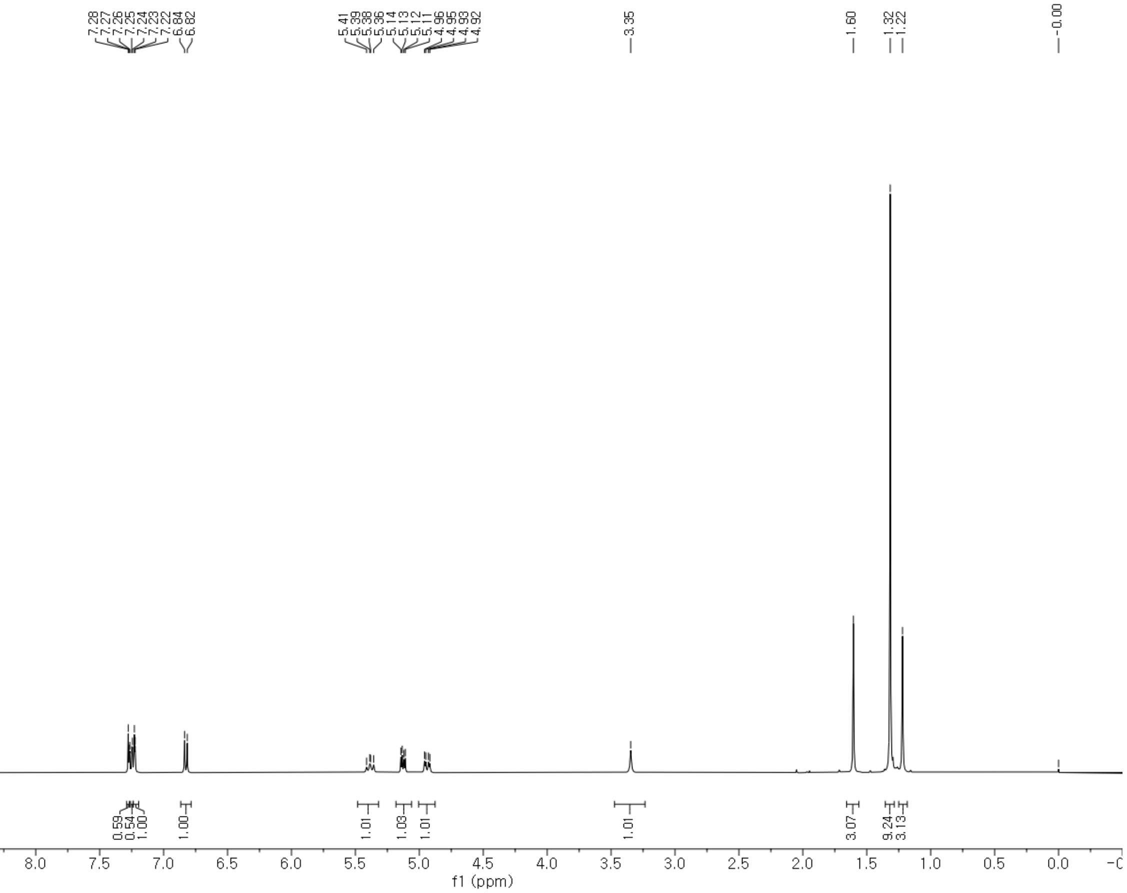

^{13}C NMR (101 MHz) in $CDCl_3$

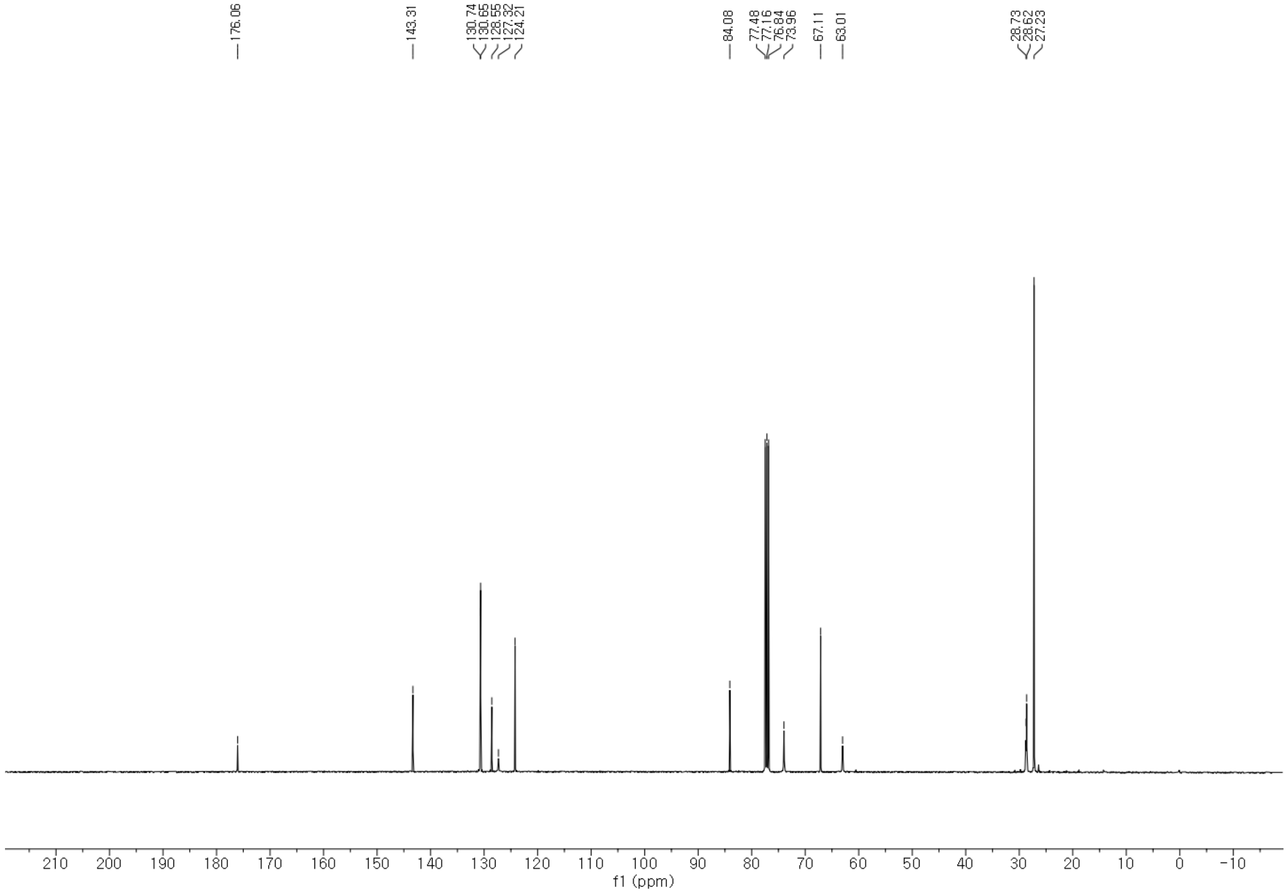

^{1}H NMR (400 MHz) in $CDCl_3$

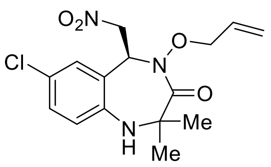

3e

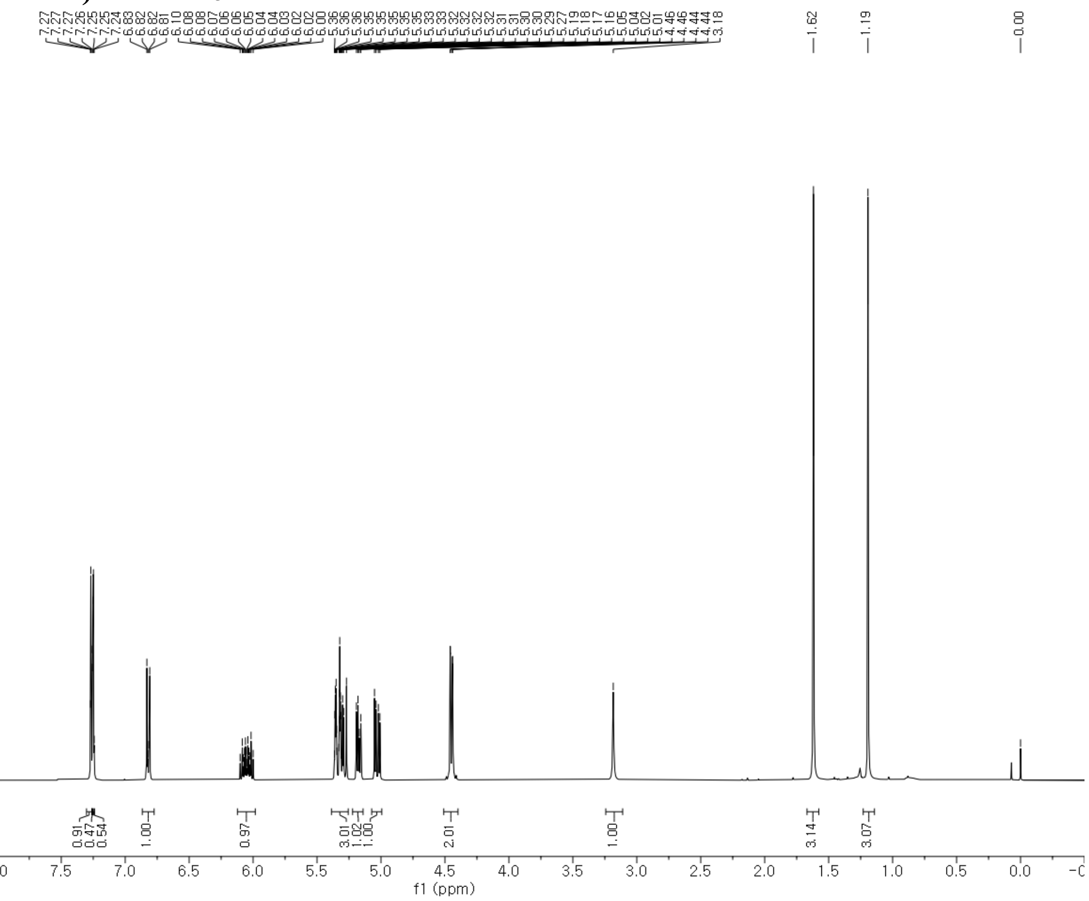

^{13}C NMR (101 MHz) in $CDCl_3$

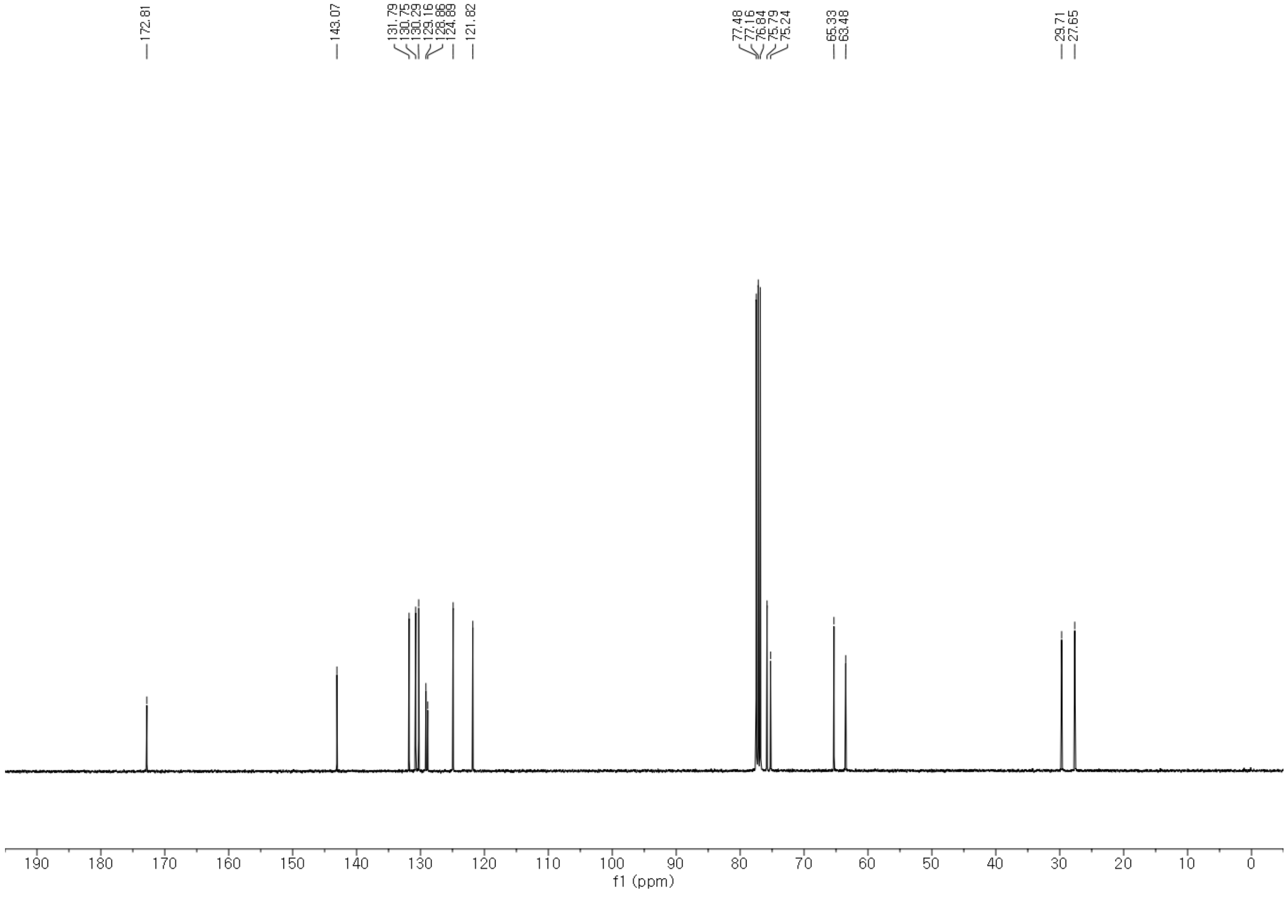

^{1}H NMR (400 MHz) in $CDCl_3$

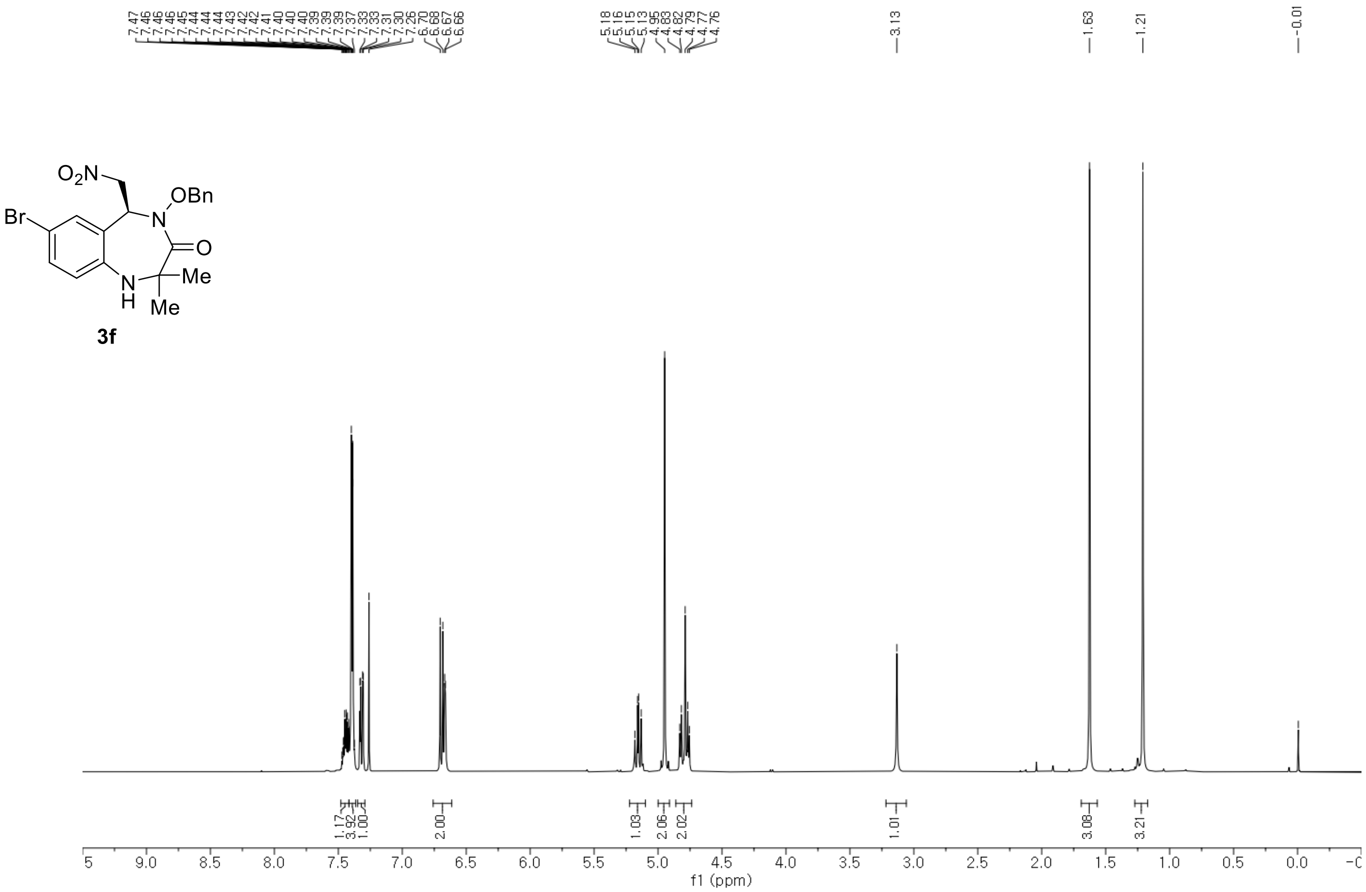

^{13}C NMR (101 MHz) in $CDCl_3$

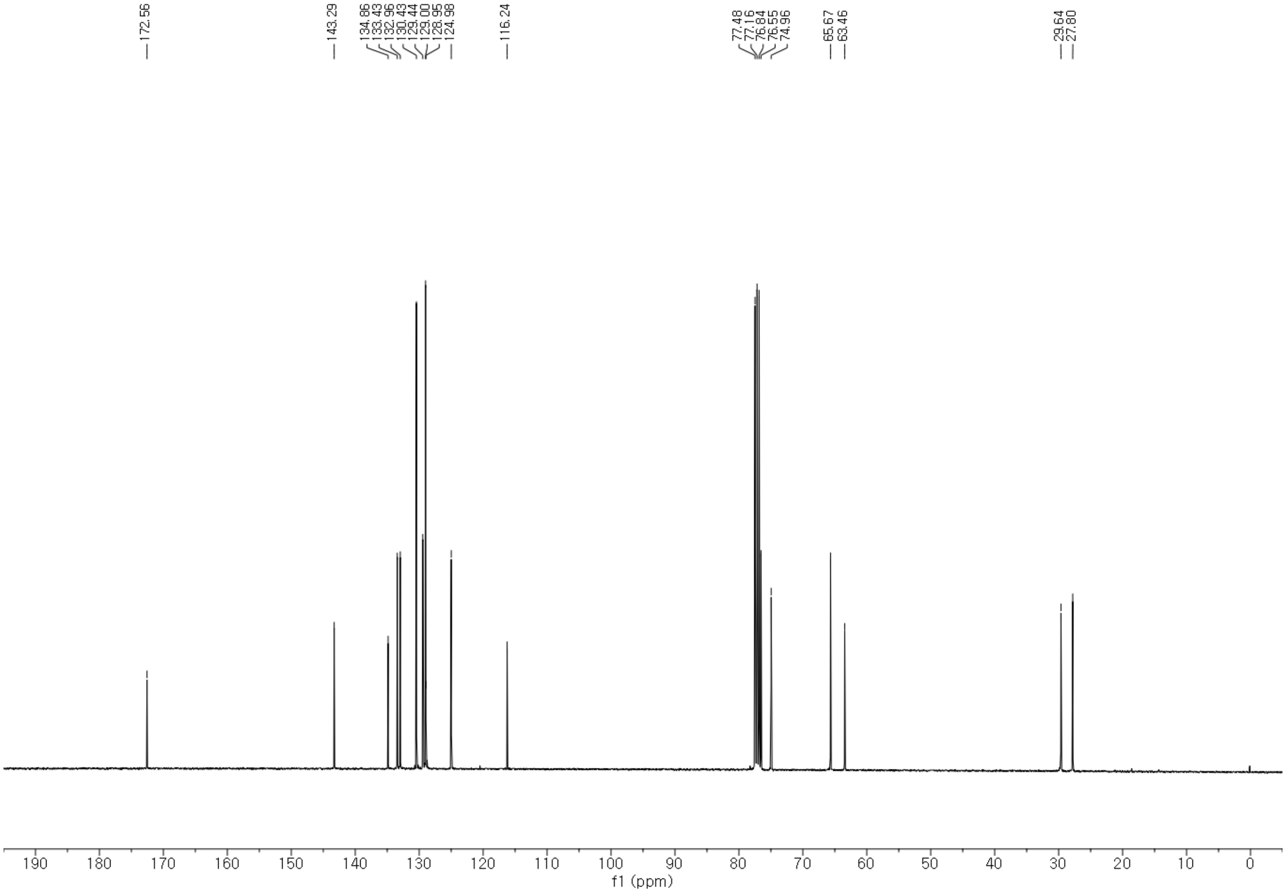

^{1}H NMR (400 MHz) in $CDCl_3$

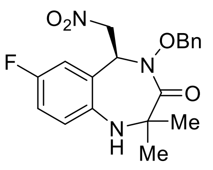

3g

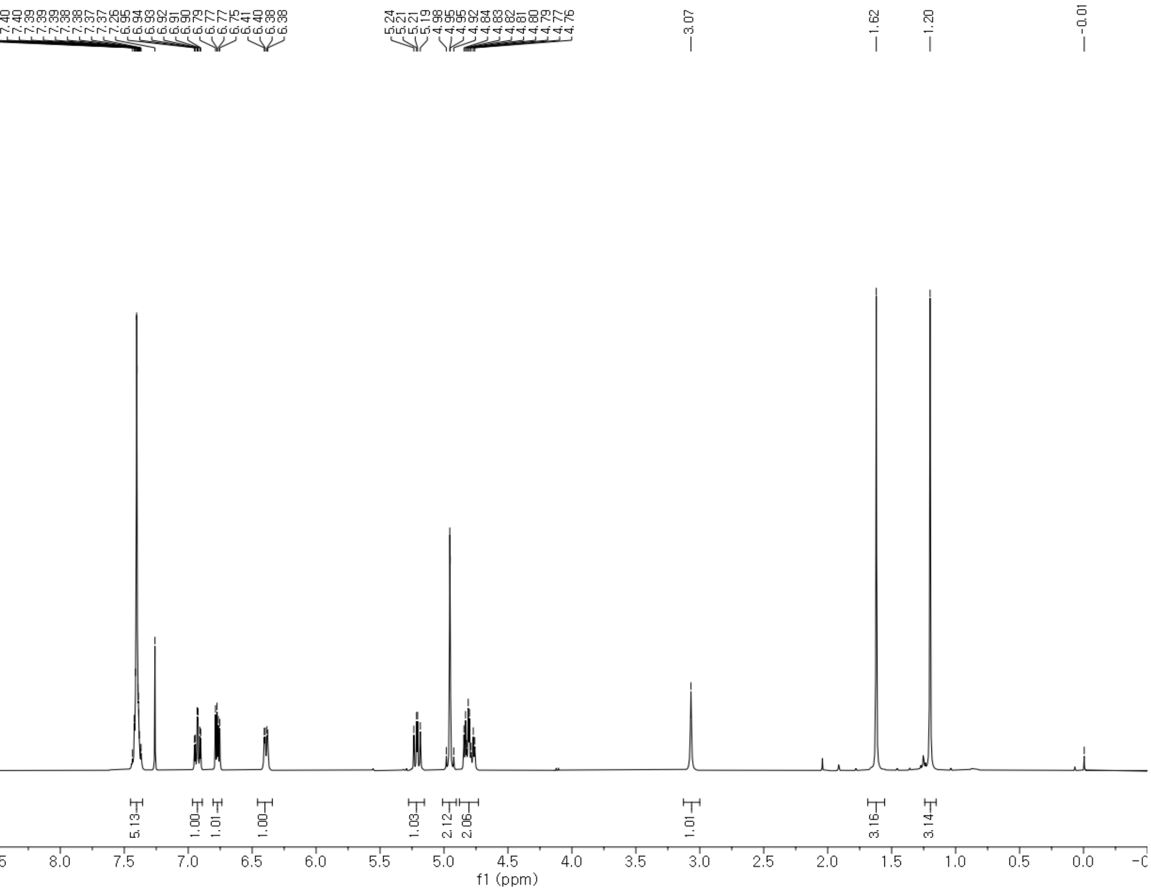

^{13}C NMR (101 MHz) in $CDCl_3$

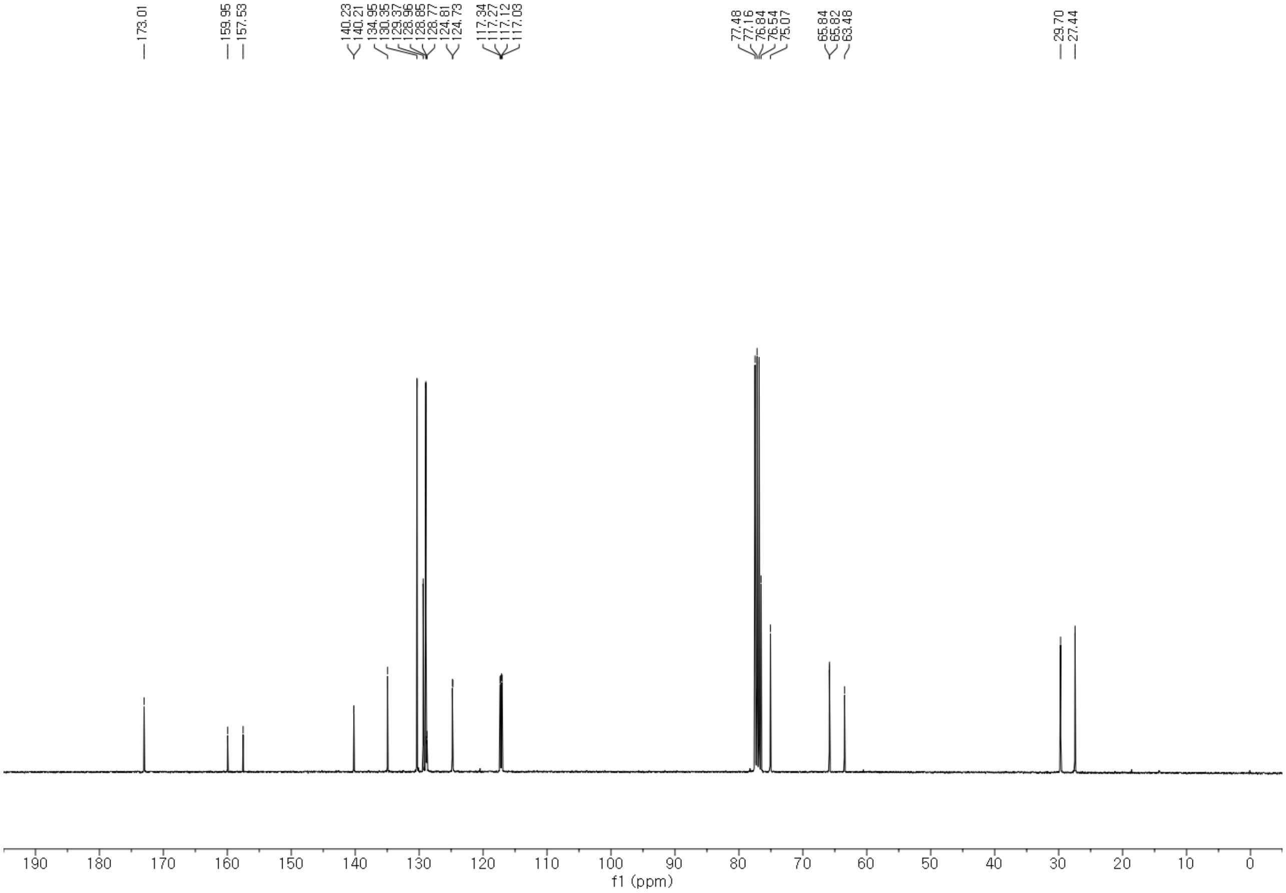

^{19}F NMR (376 MHz) in $CDCl_3$

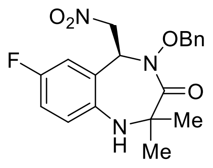

3g

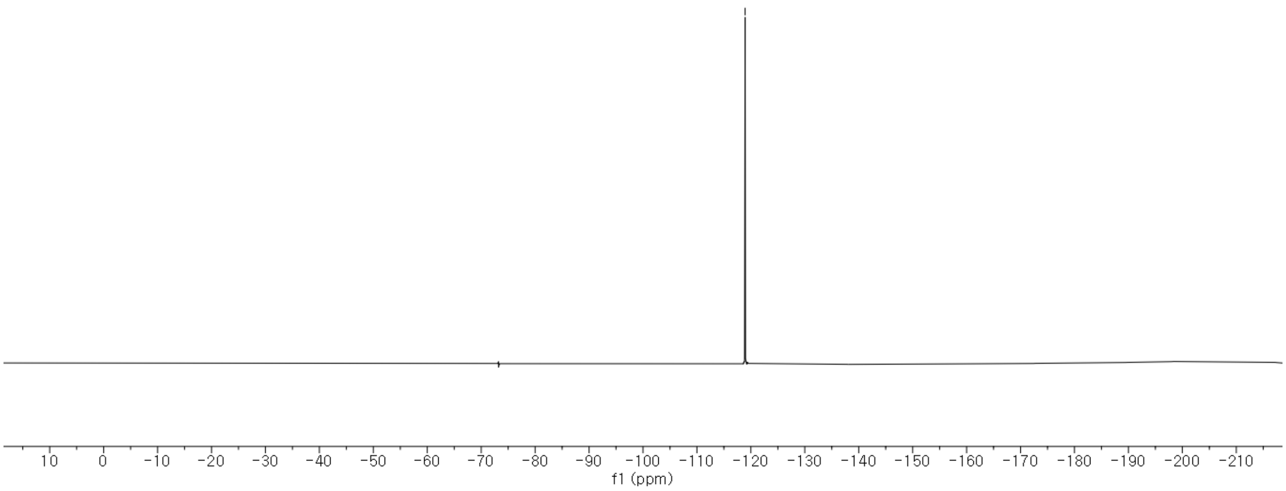

^{1}H NMR (400 MHz) in $CDCl_3$

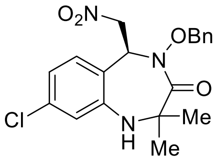

3h

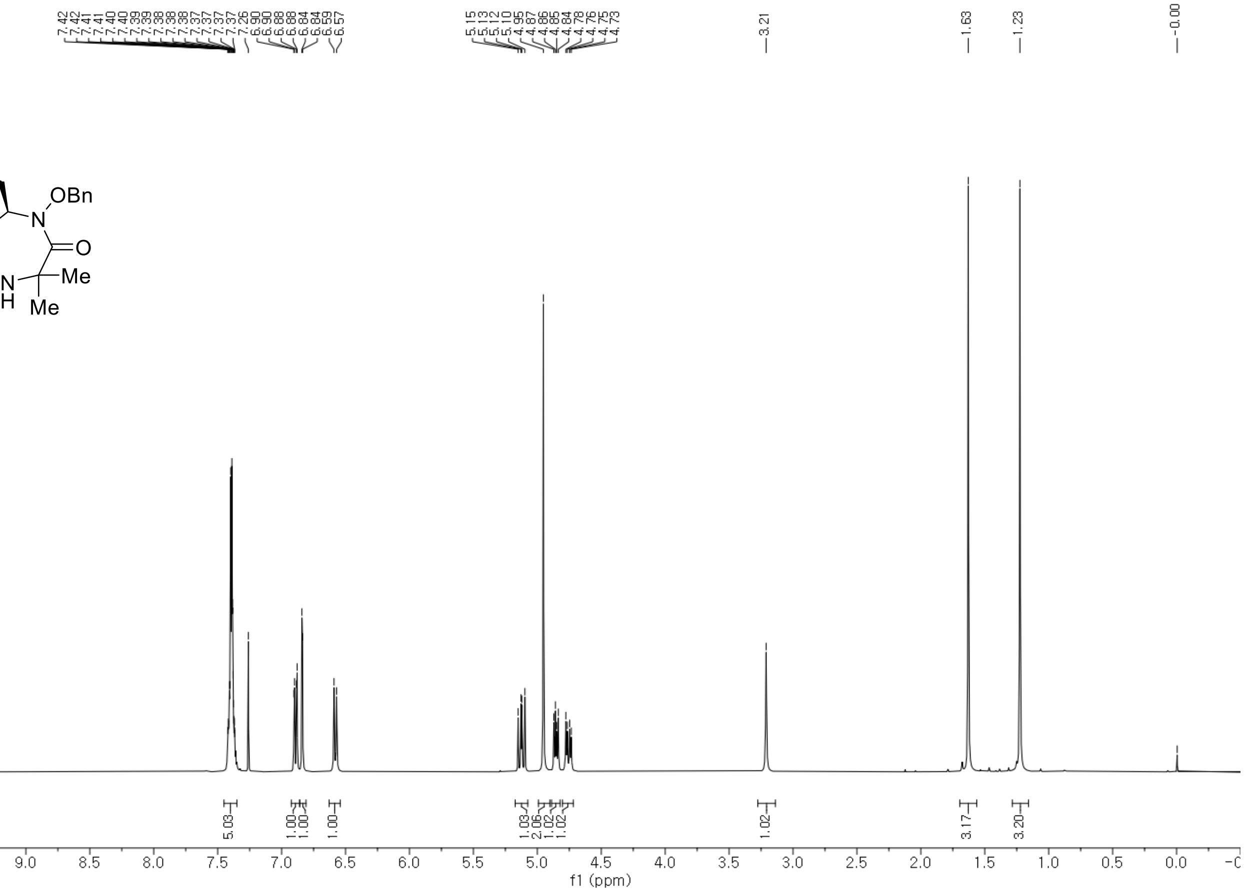

^{13}C NMR (101 MHz) in $CDCl_3$

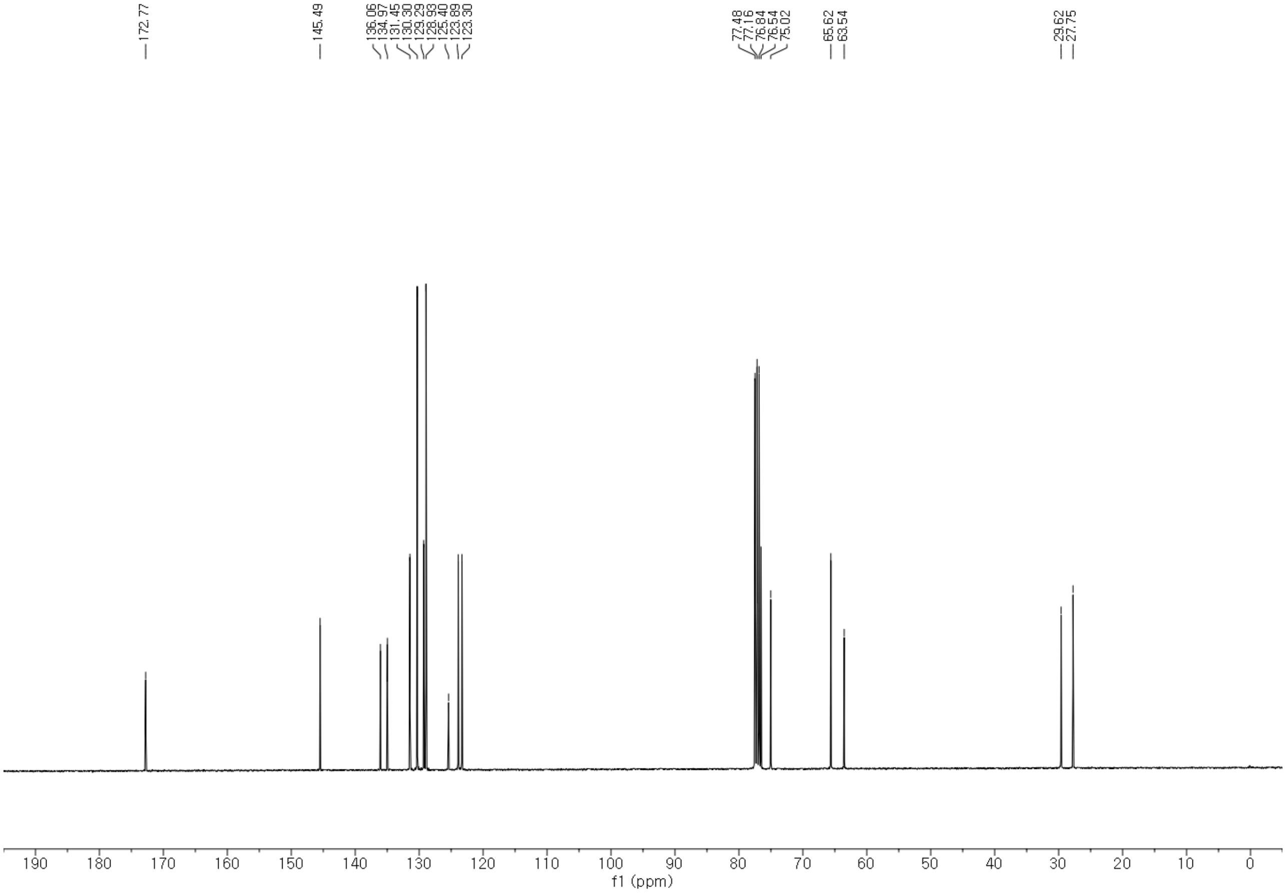

^{1}H NMR (400 MHz) in $CDCl_3$

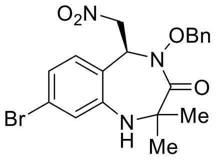

3i

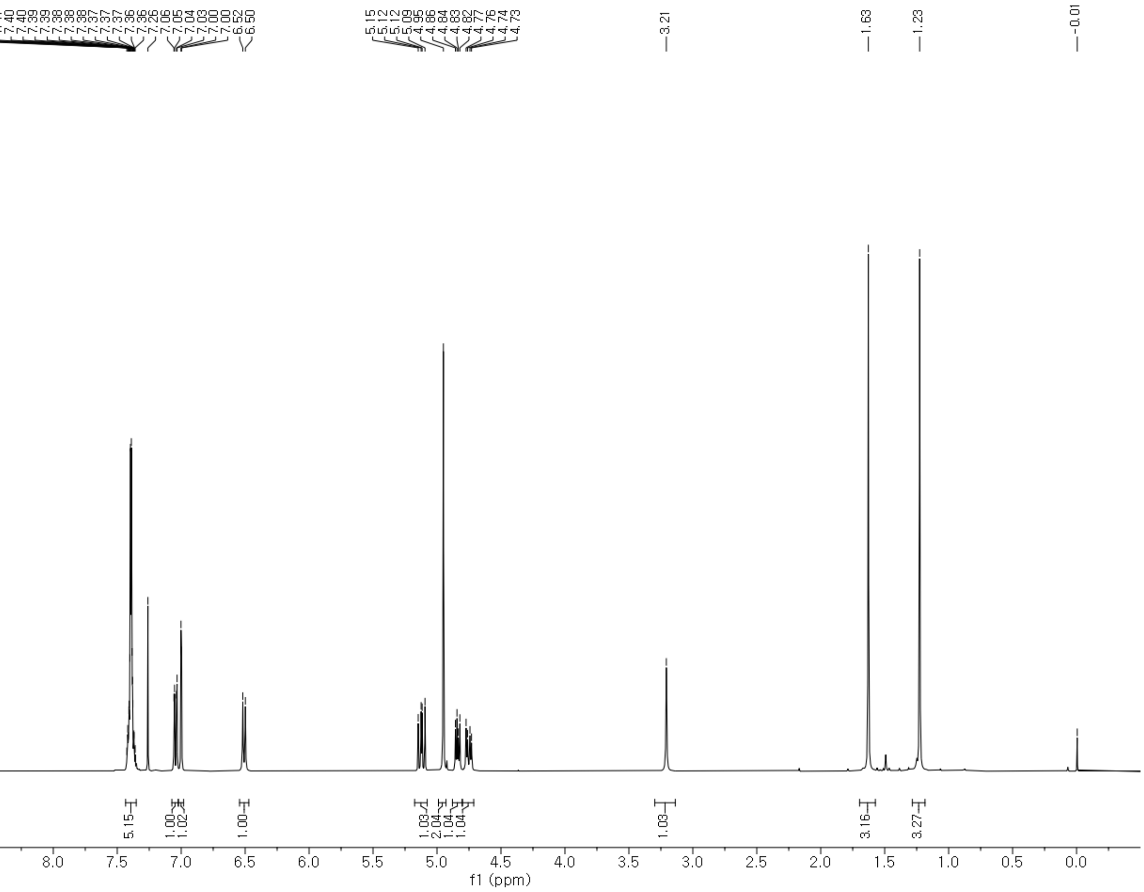

^{13}C NMR (101 MHz) in $CDCl_3$

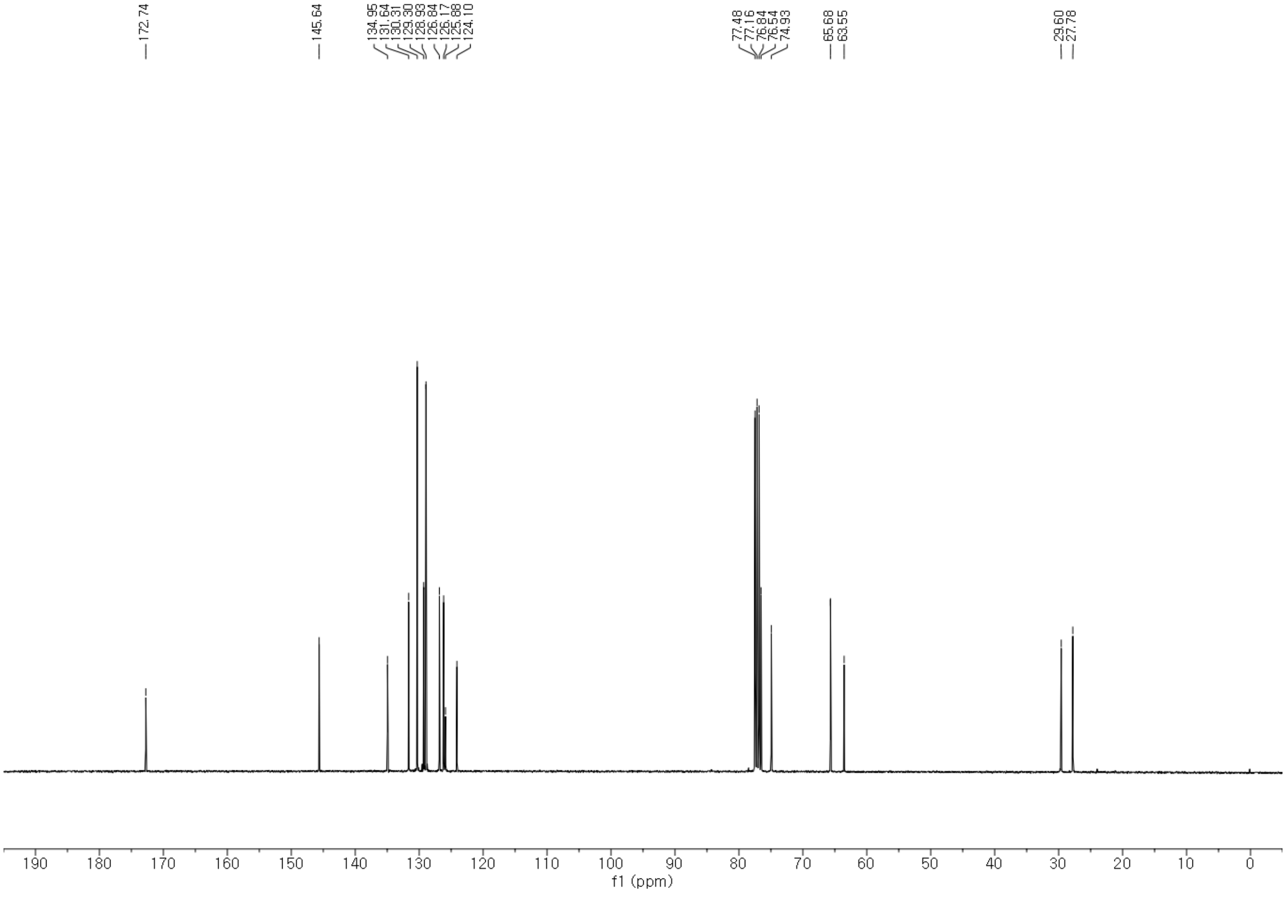

^{1}H NMR (400 MHz) in $CDCl_3$

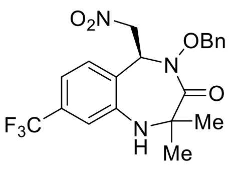

3j

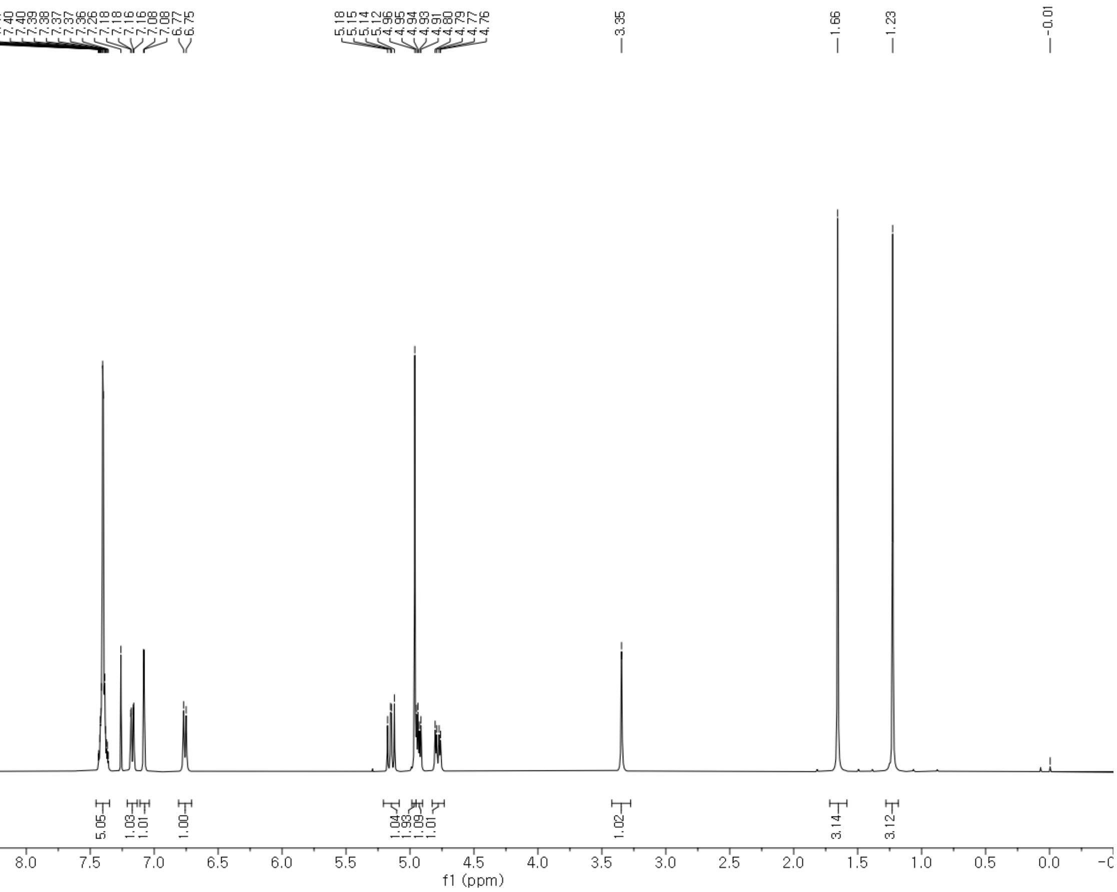

^{13}C NMR (101 MHz) in $CDCl_3$

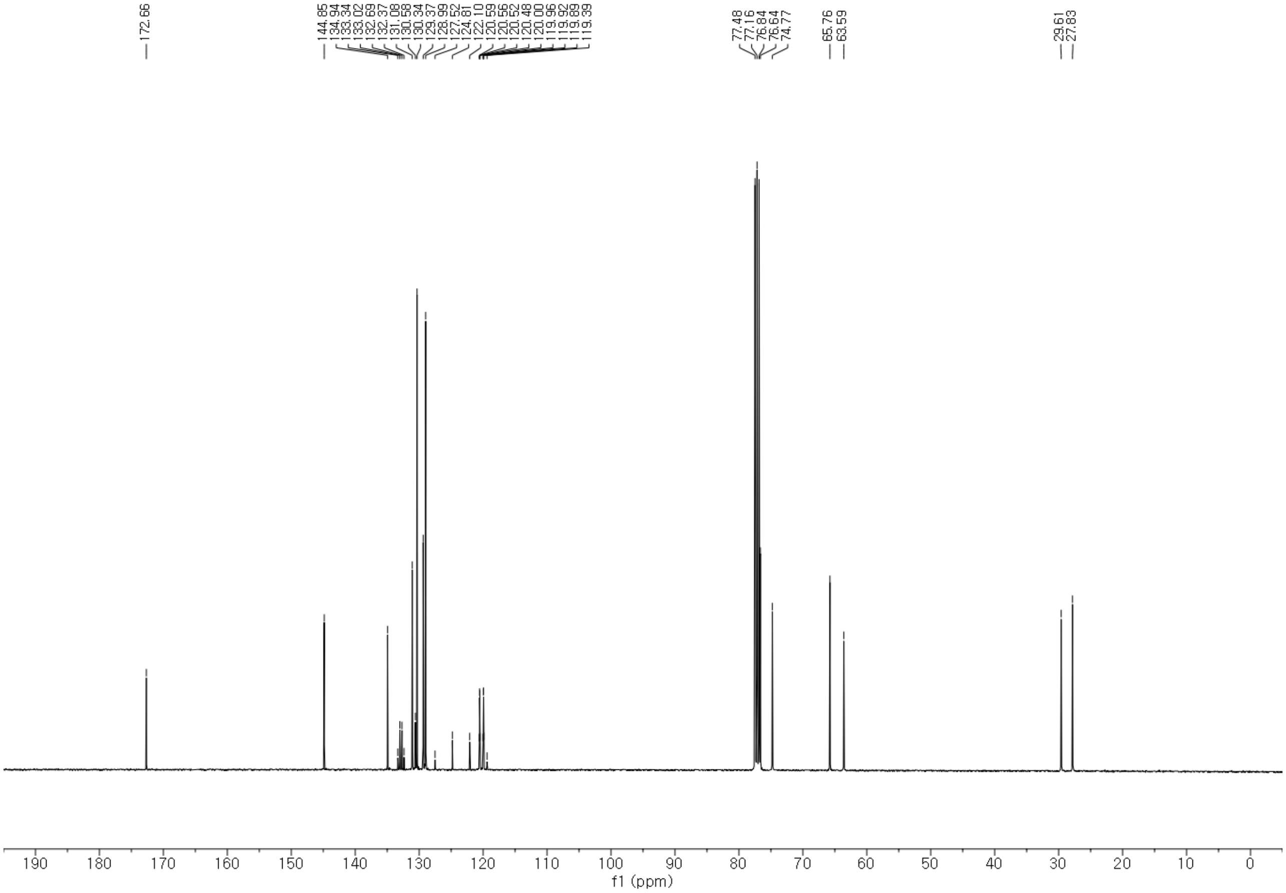

^{19}F NMR (376 MHz) in $CDCl_3$

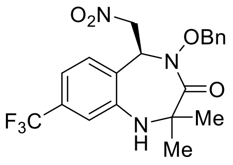

3j

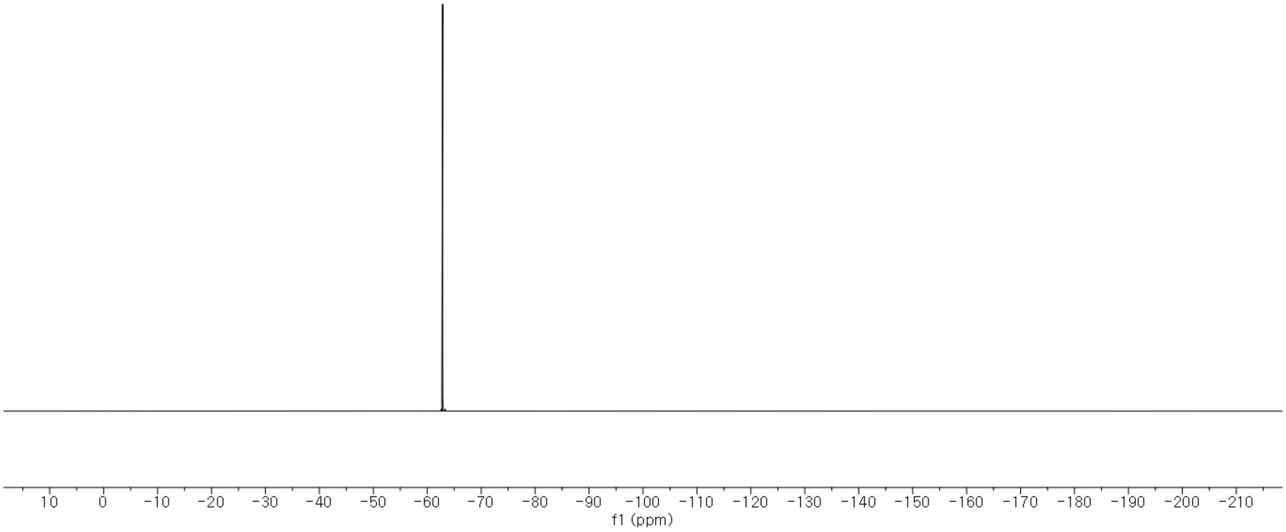

^{1}H NMR (400 MHz) in $CDCl_3$

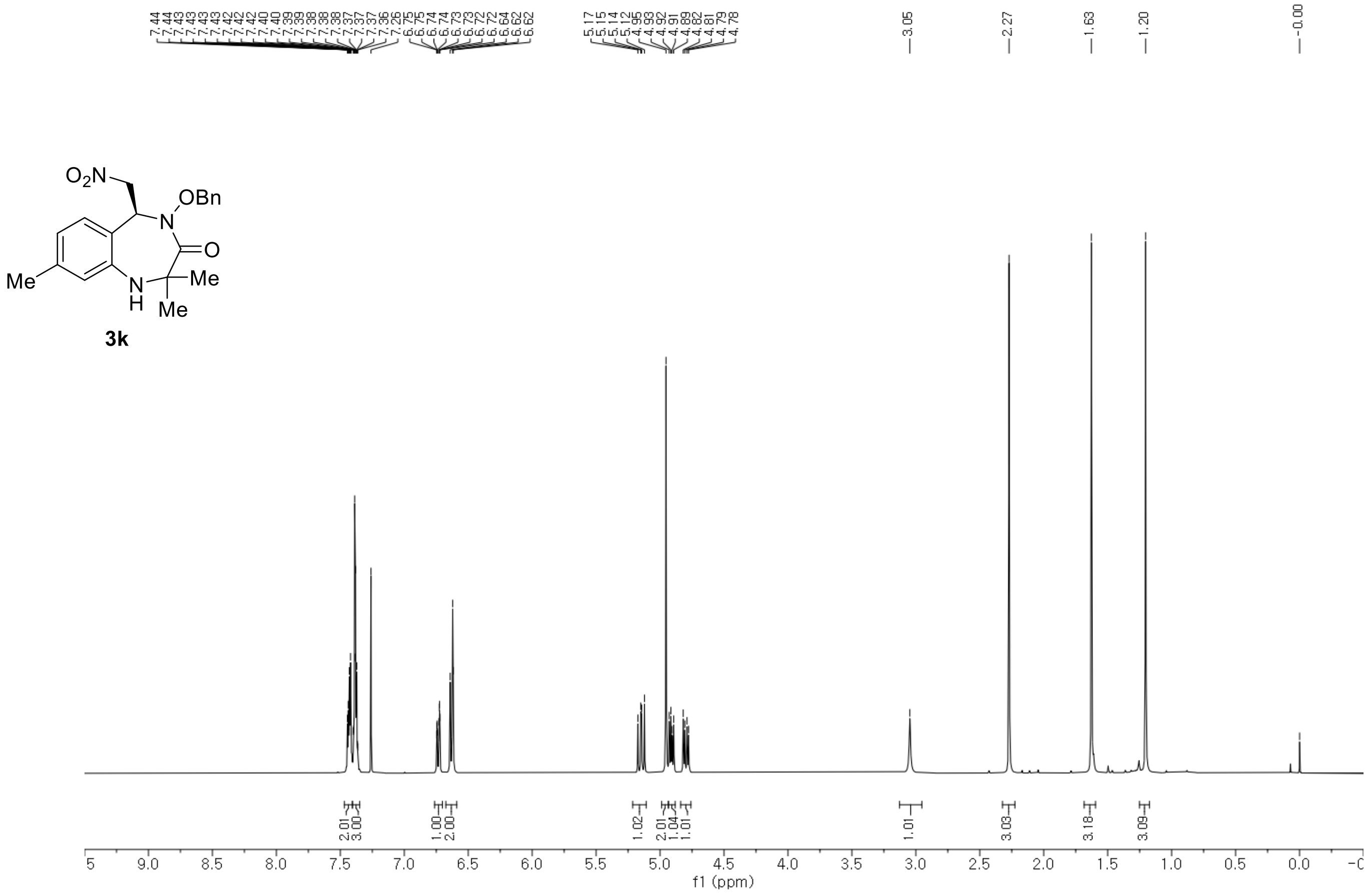

3k

^{13}C NMR (101 MHz) in $CDCl_3$

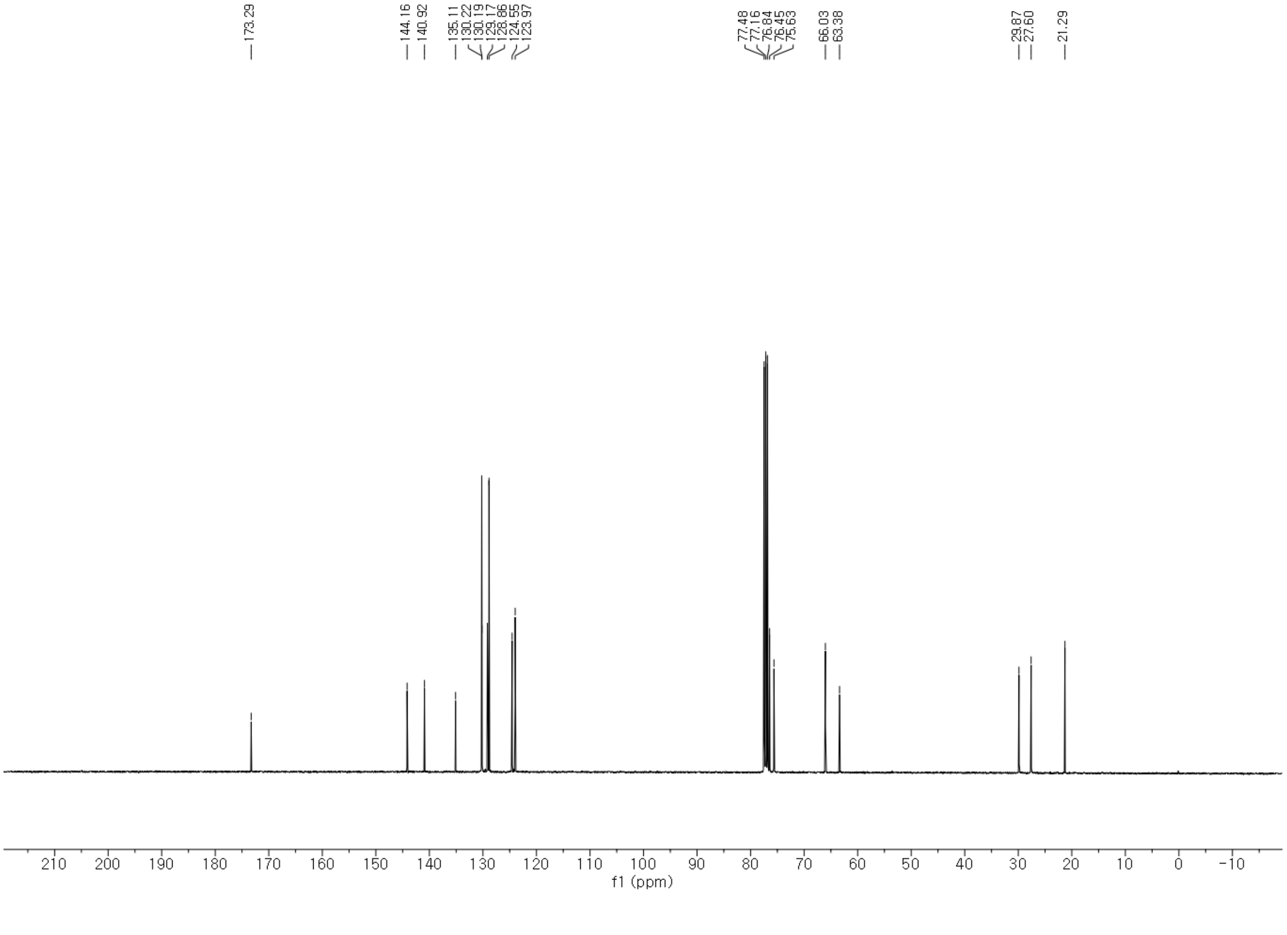

^{1}H NMR (400 MHz) in $CDCl_3$

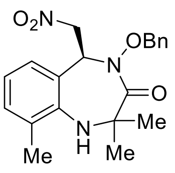

3l

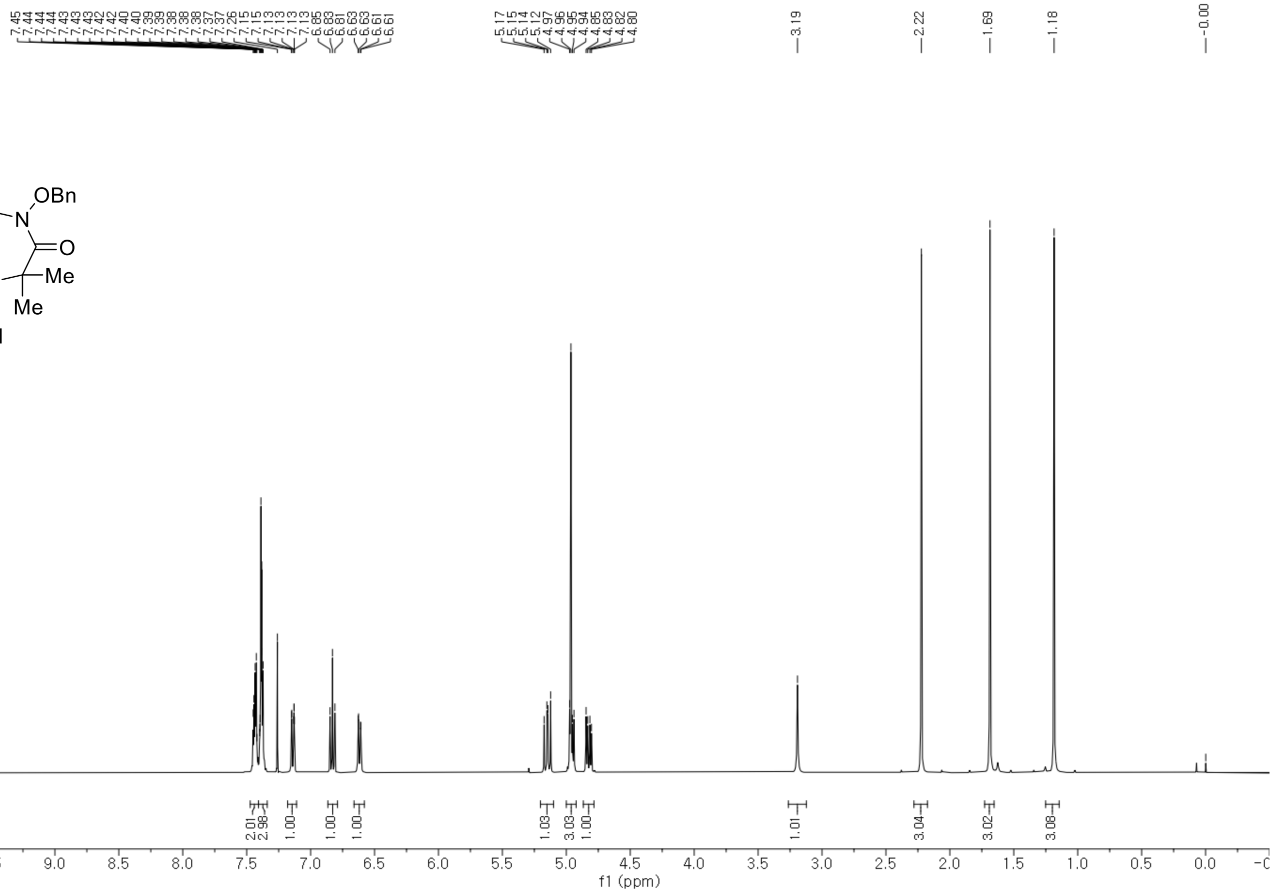

^{13}C NMR (101 MHz) in $CDCl_3$

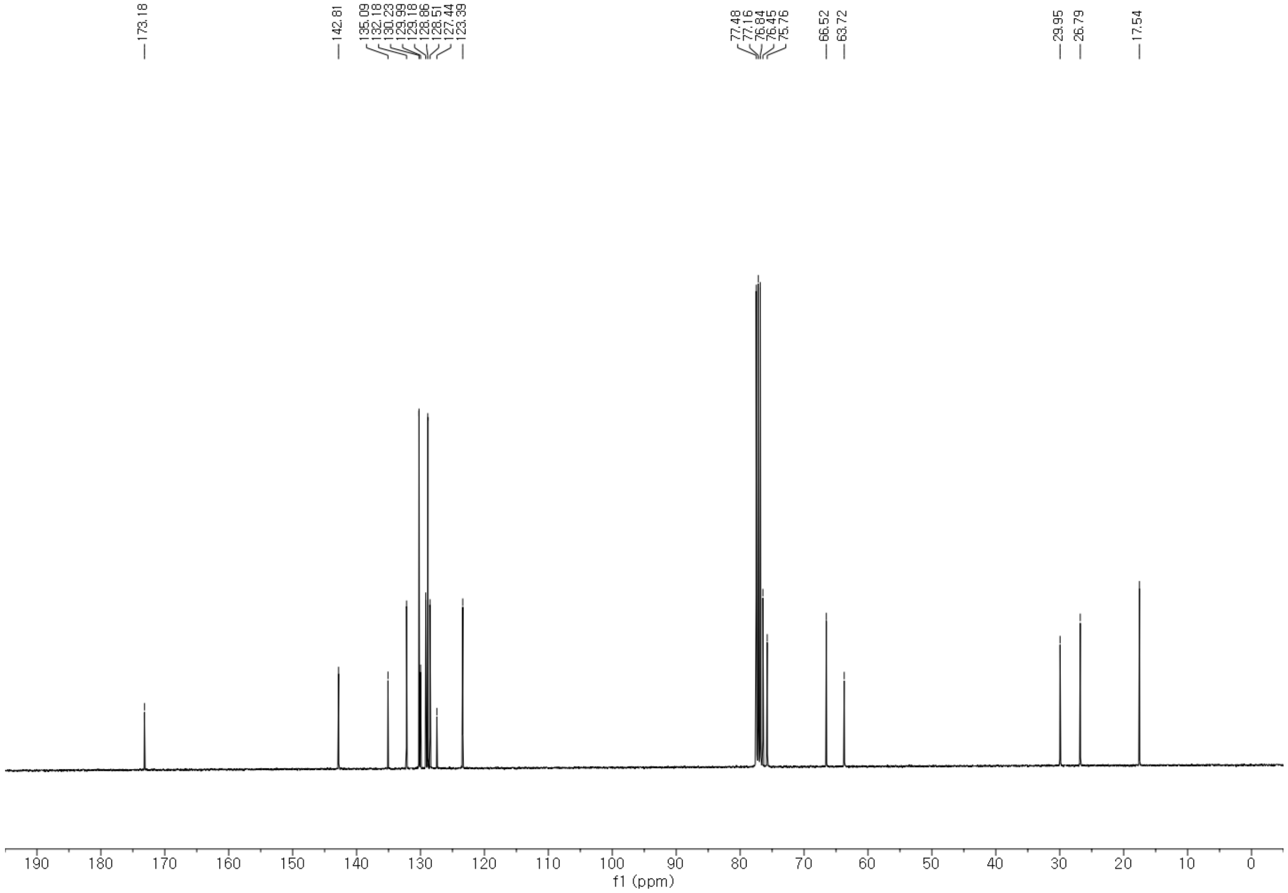

^{1}H NMR (400 MHz) in $CDCl_3$

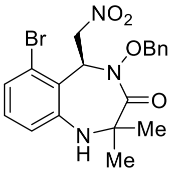

3m

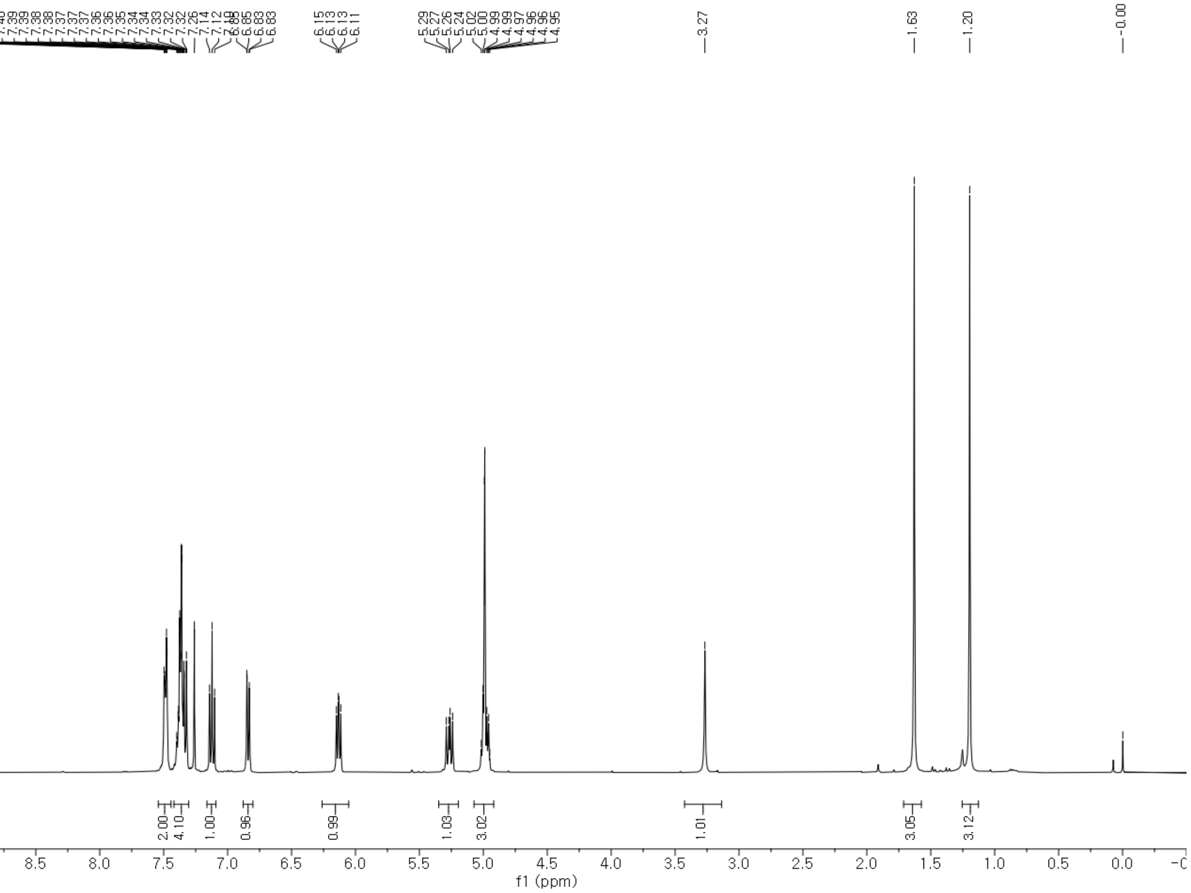

^{13}C NMR (101 MHz) in $CDCl_3$

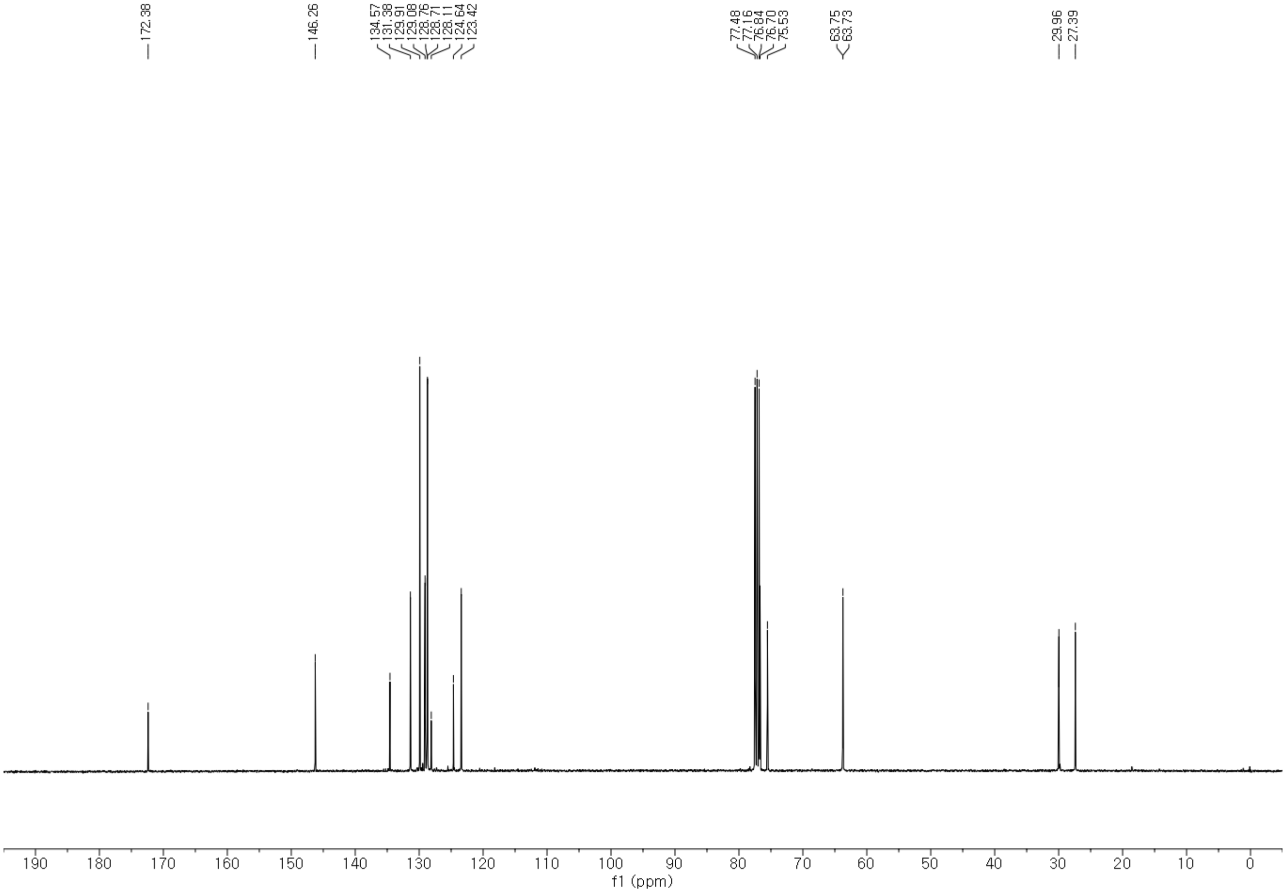

^{1}H NMR (400 MHz) in $CDCl_3$

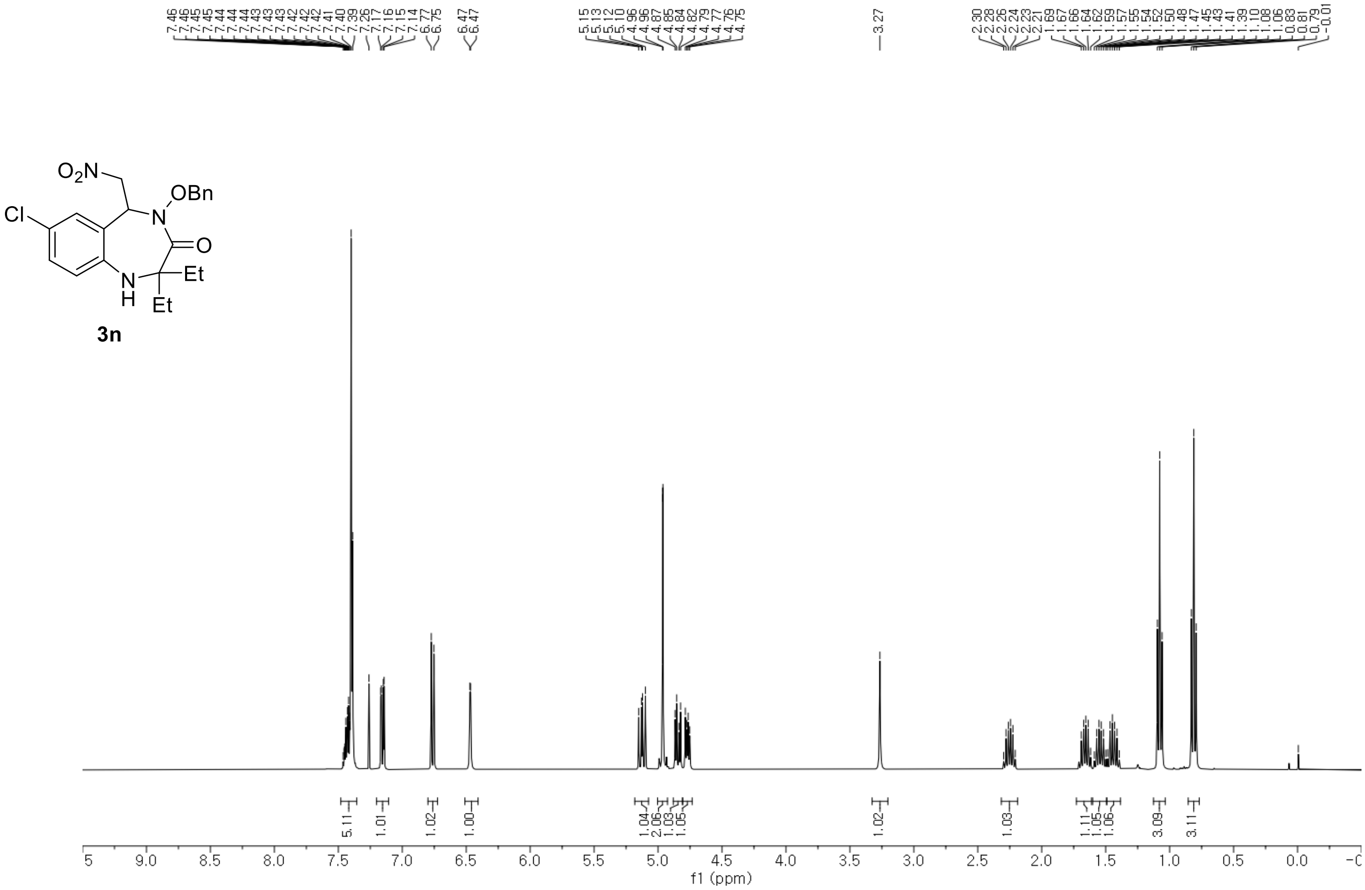

^{13}C NMR (101 MHz) in $CDCl_3$

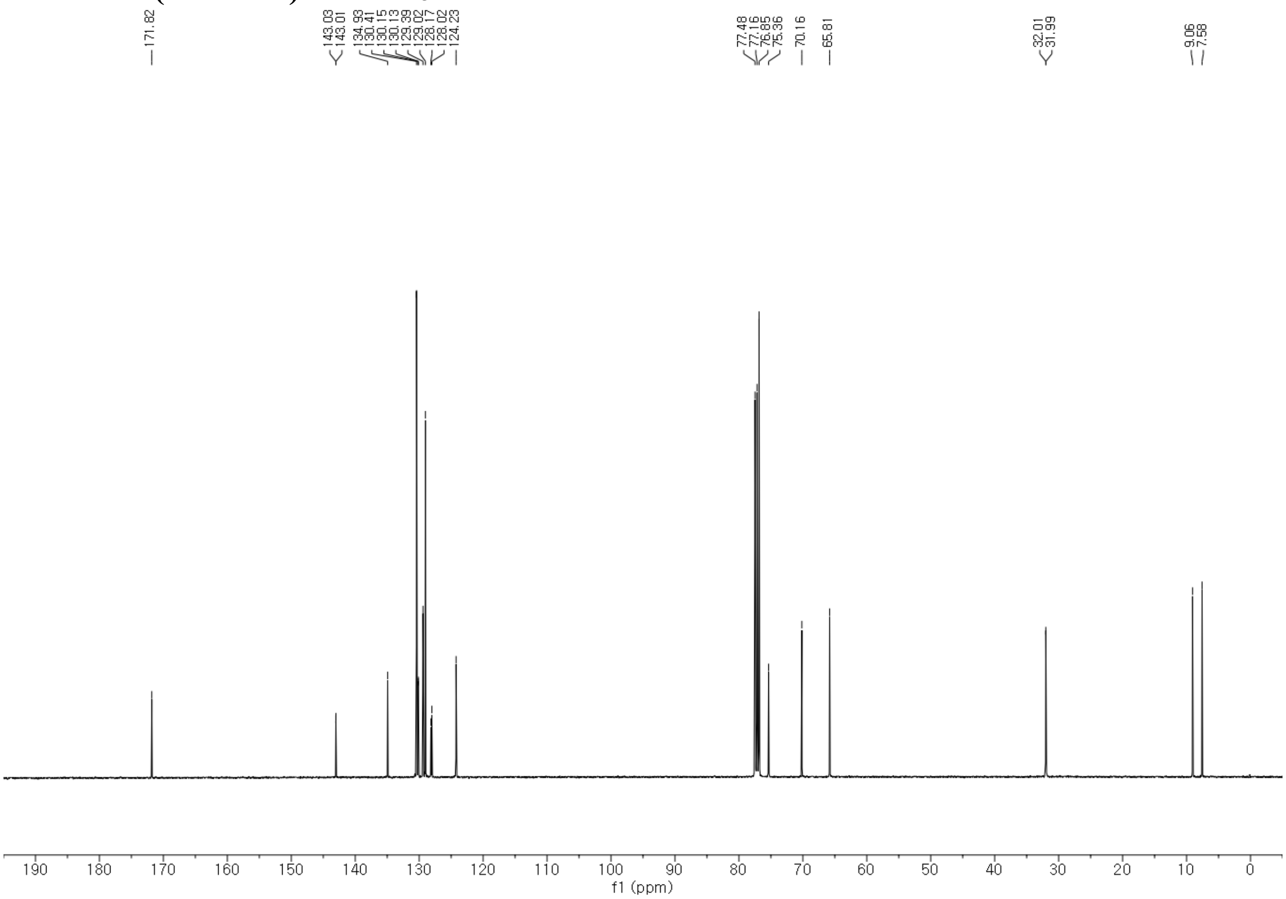

^{1}H NMR (400 MHz) in $CDCl_3$

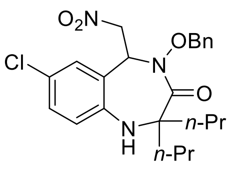

3o

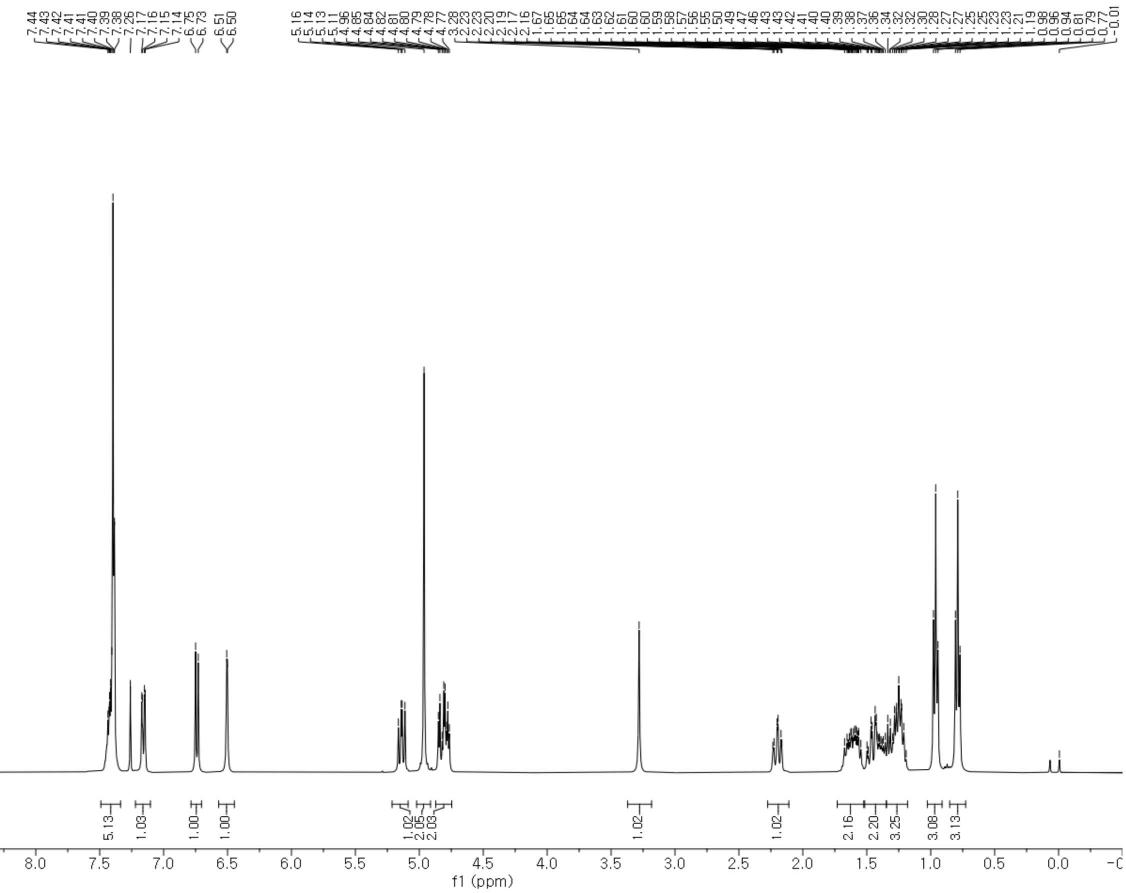

^{13}C NMR (101 MHz) in $CDCl_3$

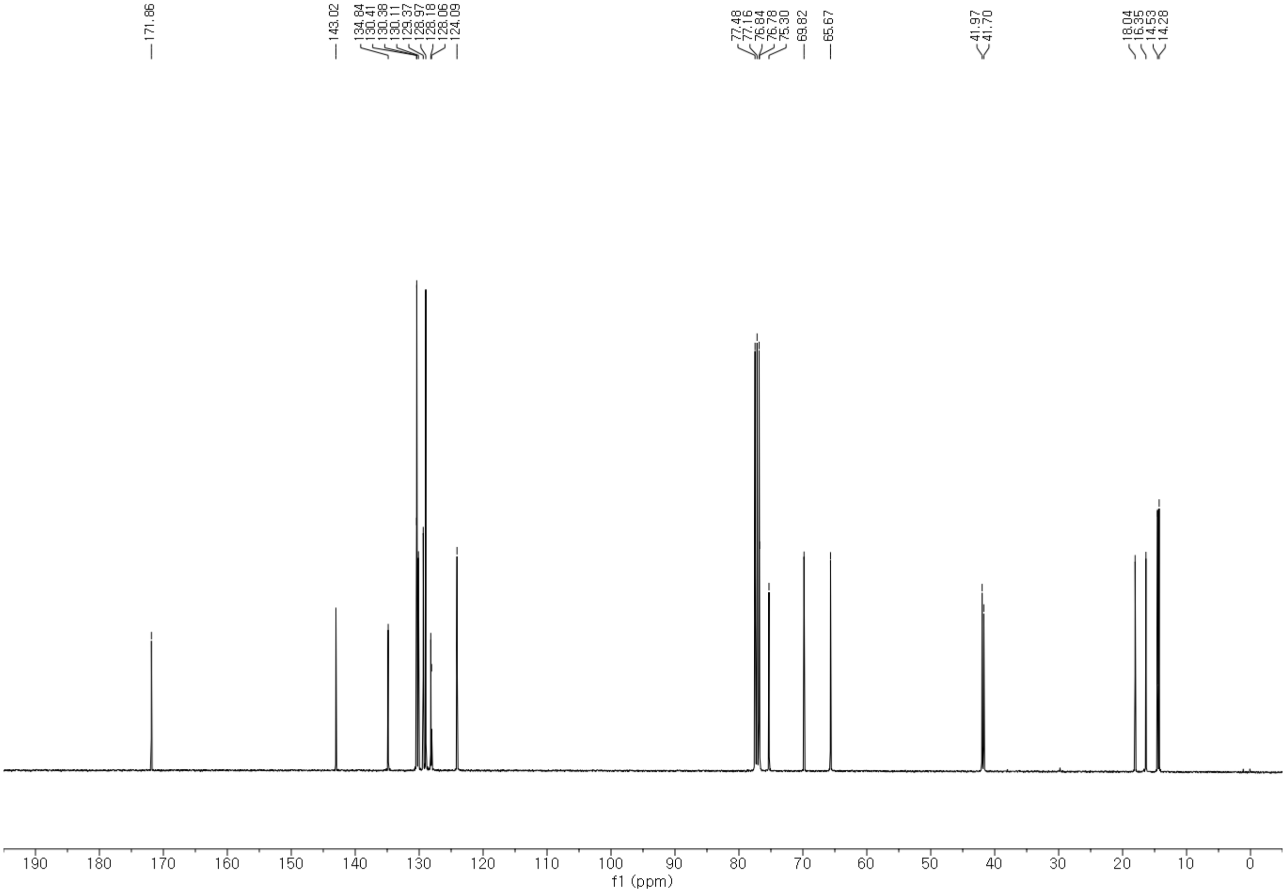

^{1}H NMR (400 MHz) in $CDCl_3$

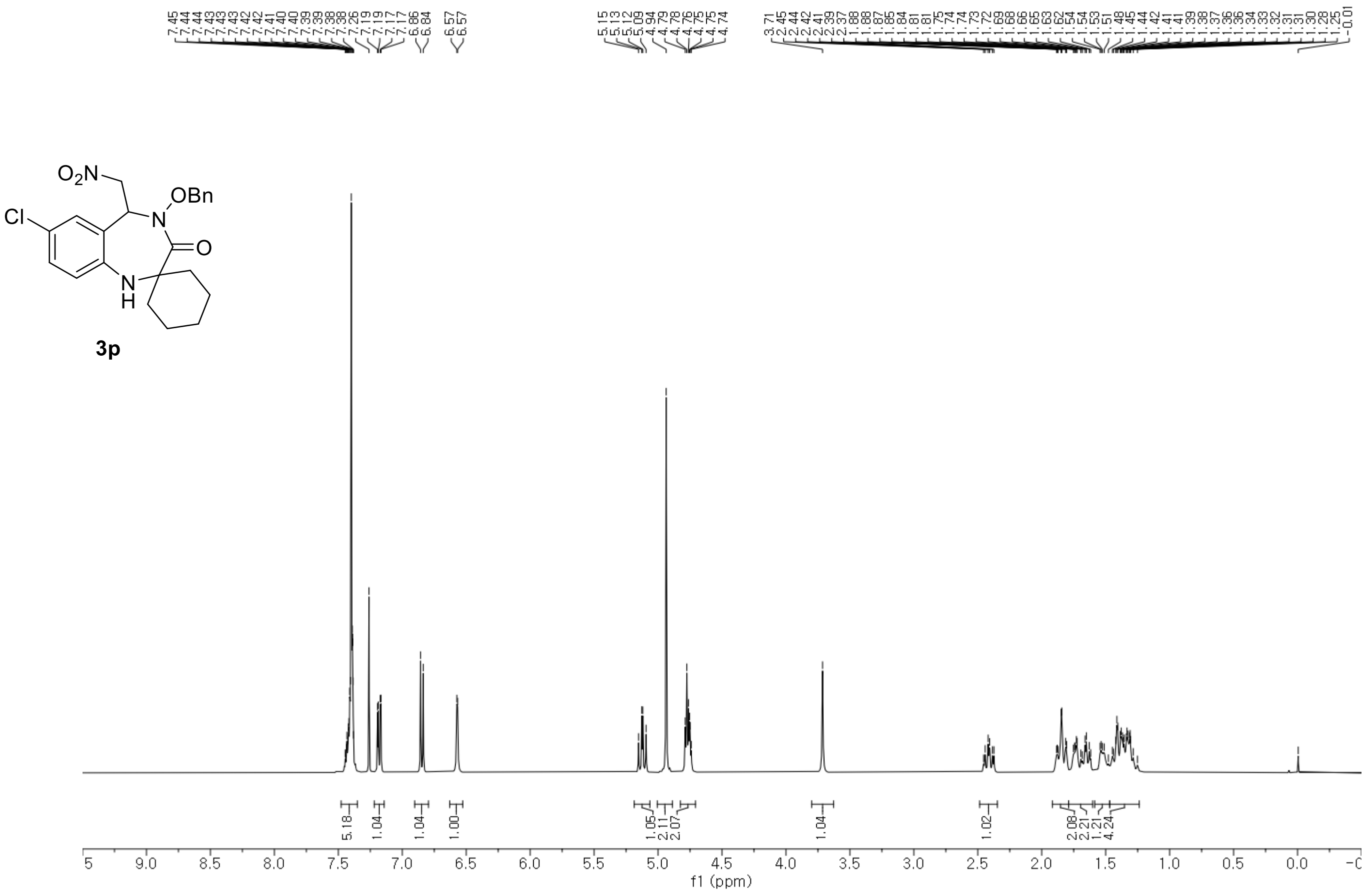

^{13}C NMR (101 MHz) in $CDCl_3$

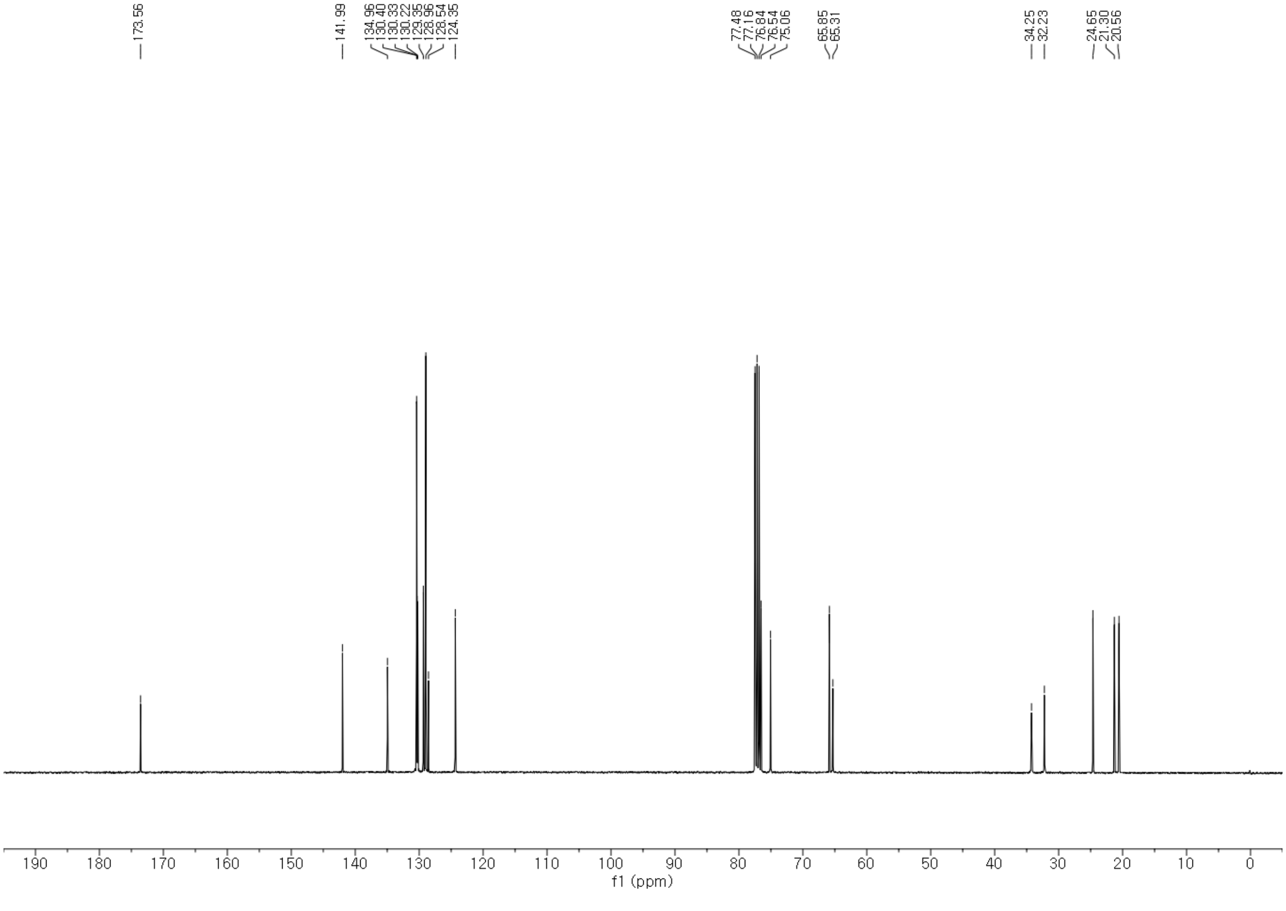

^{1}H NMR (400 MHz) in $CDCl_3$

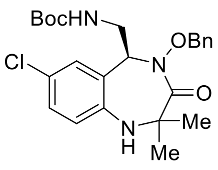

4

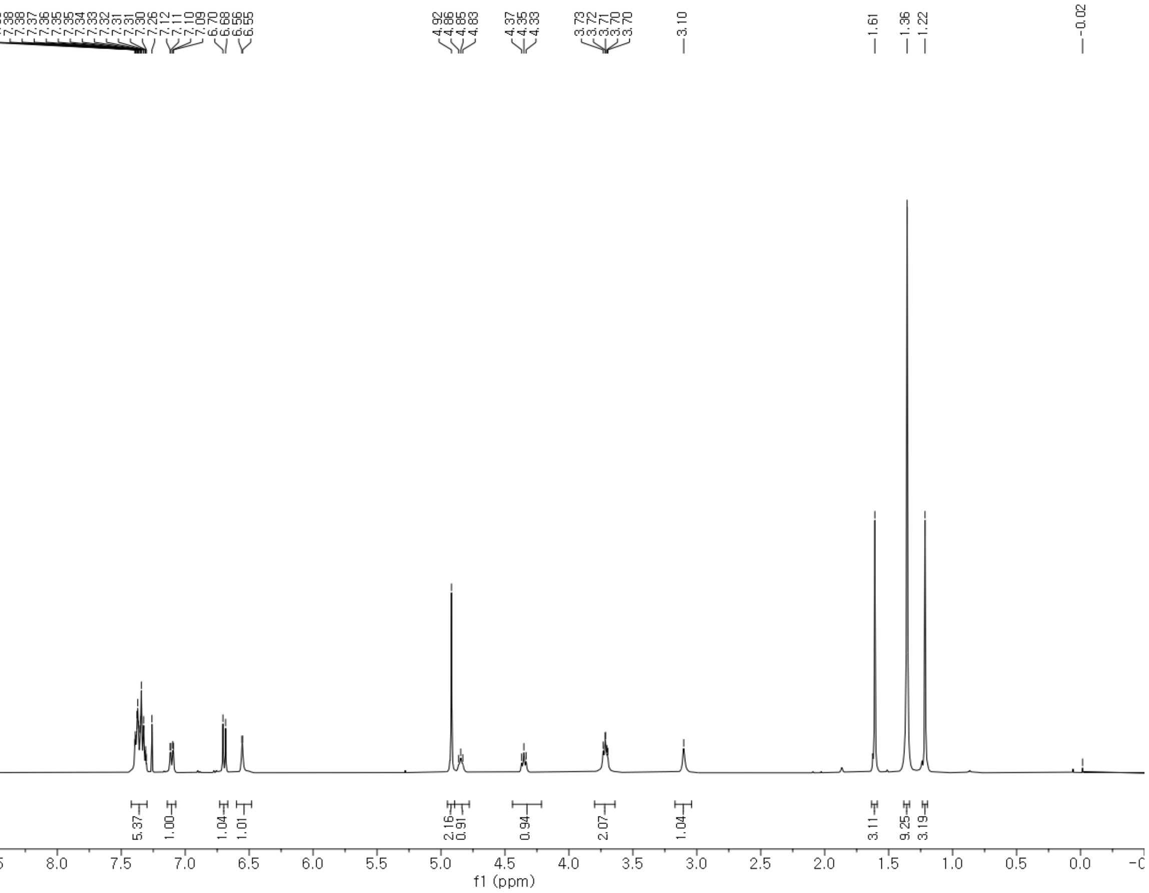

^{13}C NMR (101 MHz) in $CDCl_3$

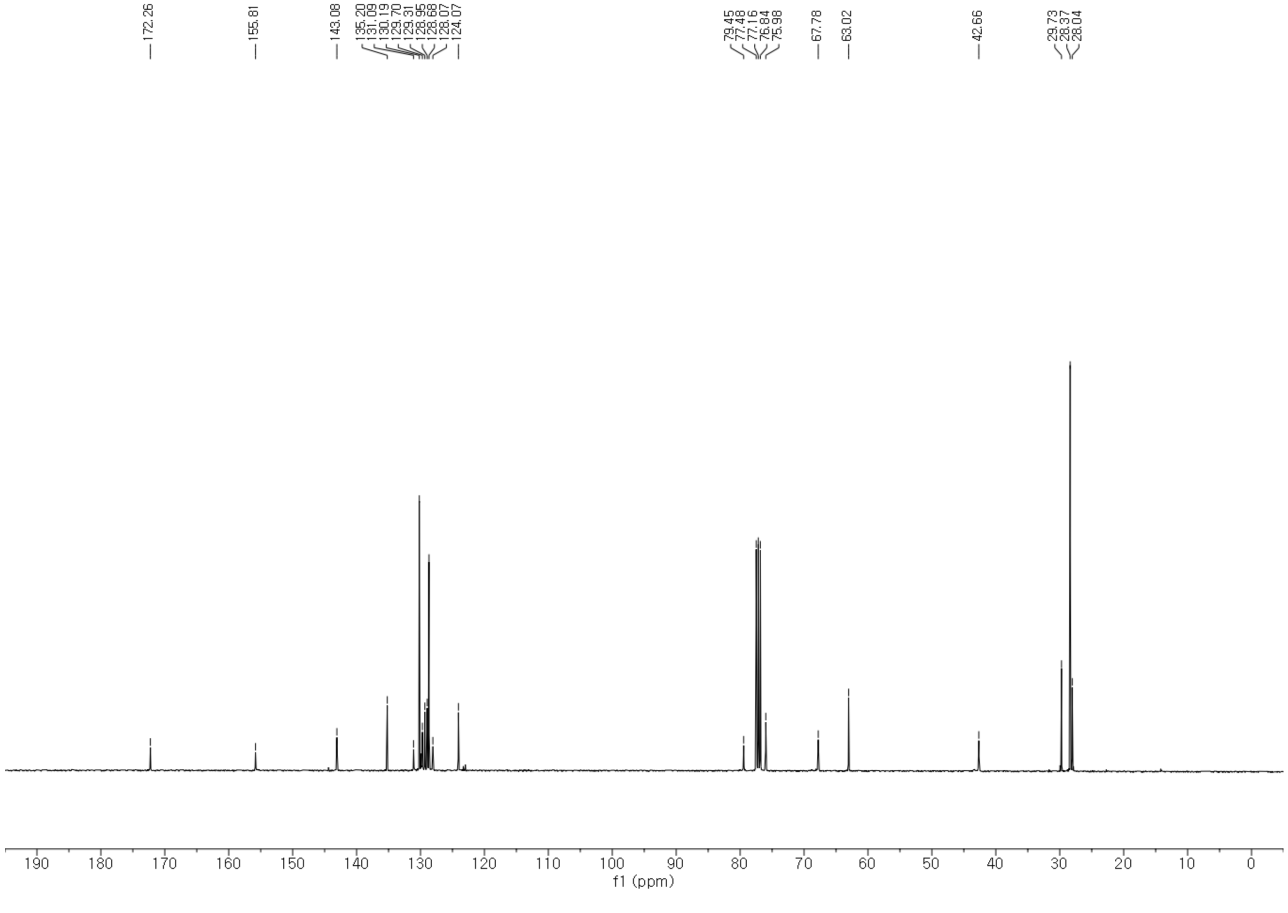

^{1}H NMR (400 MHz) in $CDCl_3$

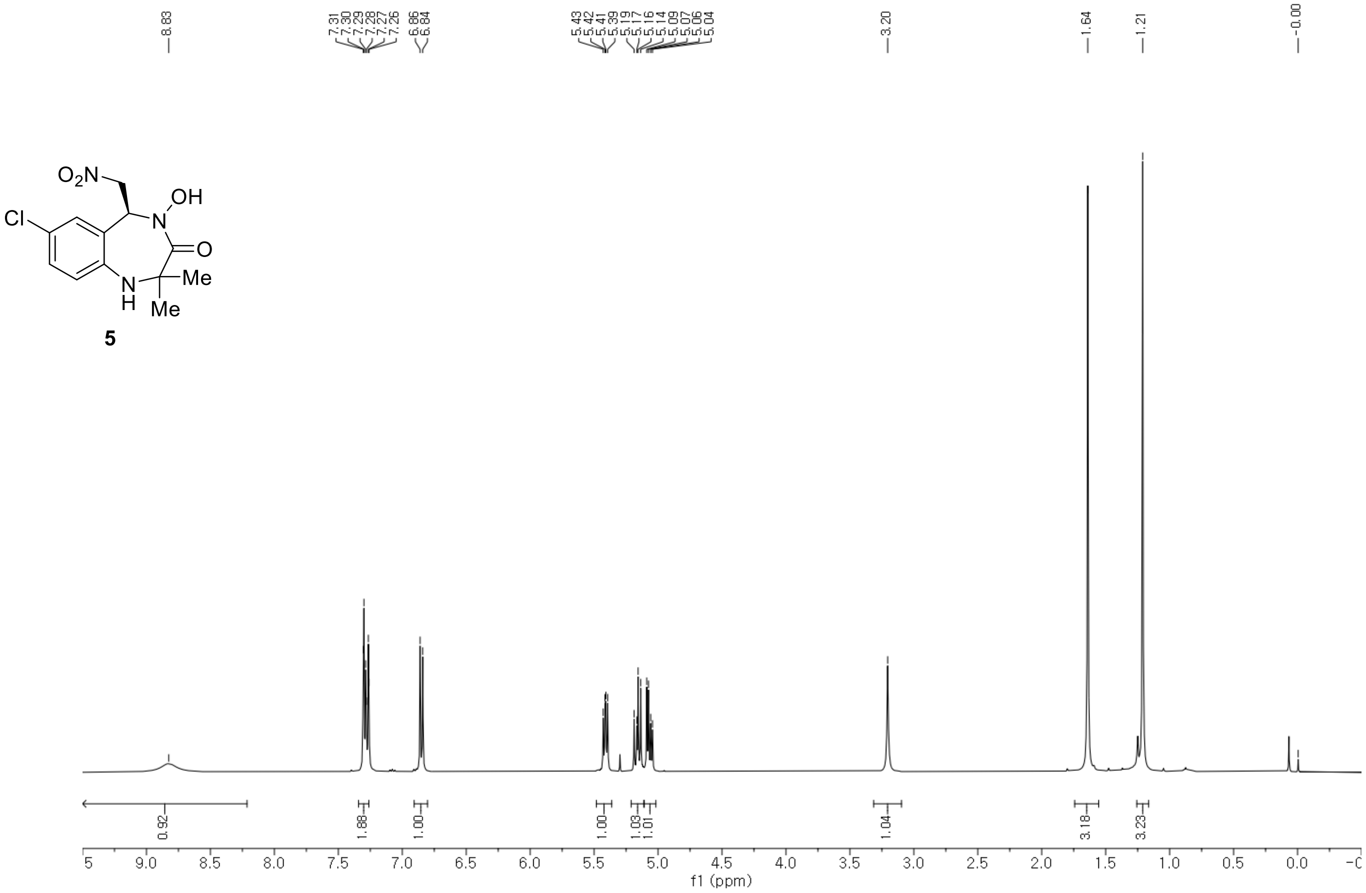

^{13}C NMR (101 MHz) in $CDCl_3$

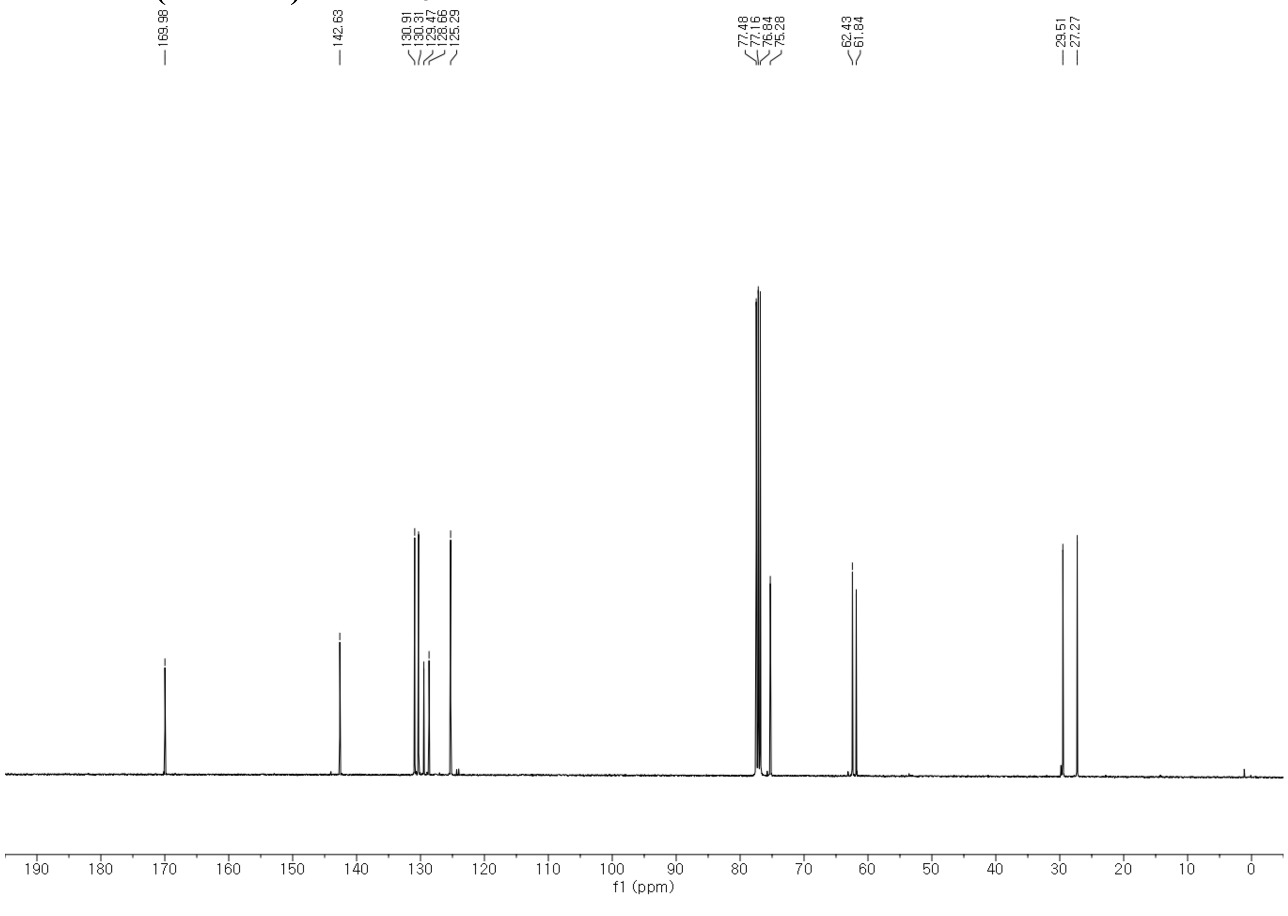

HPLC analysis

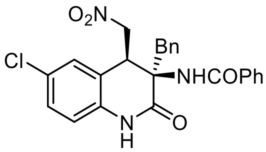

3aa

racemic

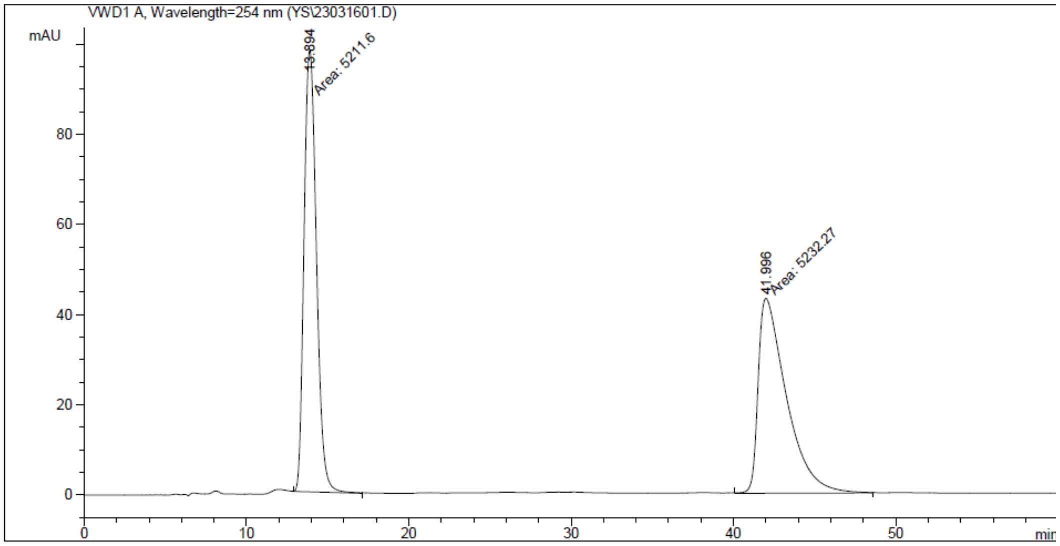

Area Percent Report

Peak #	RetTime [min]	Type	Width [min]	Area mAU *s	Height [mAU]	Area %
1	13.894	MM	0.8859	5211.60303	98.04404	49.9010
2	41.996	MM	2.0150	5232.27197	43.27857	50.0990
Totals :				1.04439e4	141.32262	

enantiomerically enriched

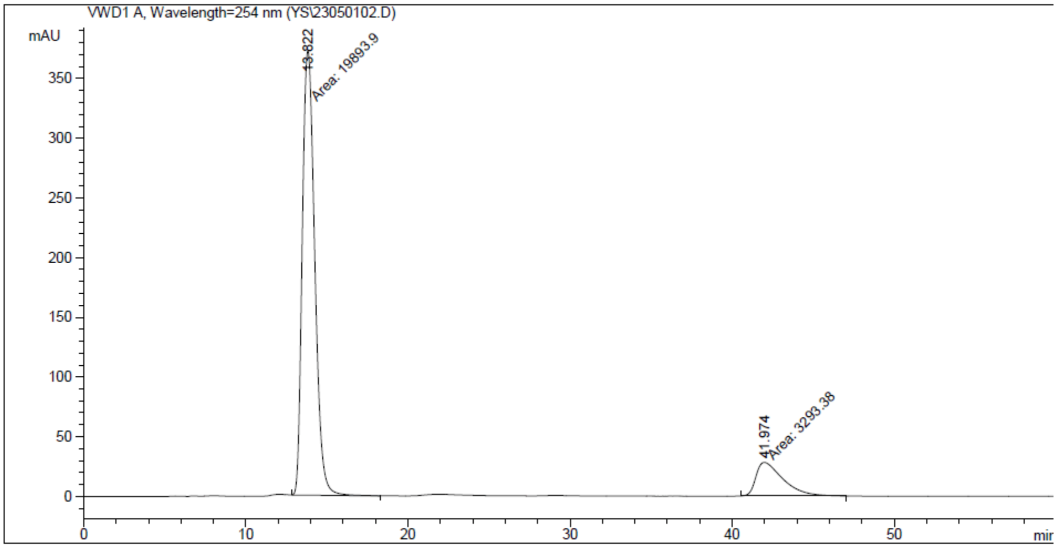

Area Percent Report

Peak #	RetTime [min]	Type	Width [min]	Area mAU *s	Height [mAU]	Area %
1	13.822	MM	0.8899	1.98939e4	372.59503	85.7966
2	41.974	MM	1.9455	3293.37964	28.21436	14.2034
Totals :				2.31873e4	400.80939	

HPLC analysis

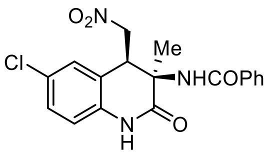

3ab

racemic

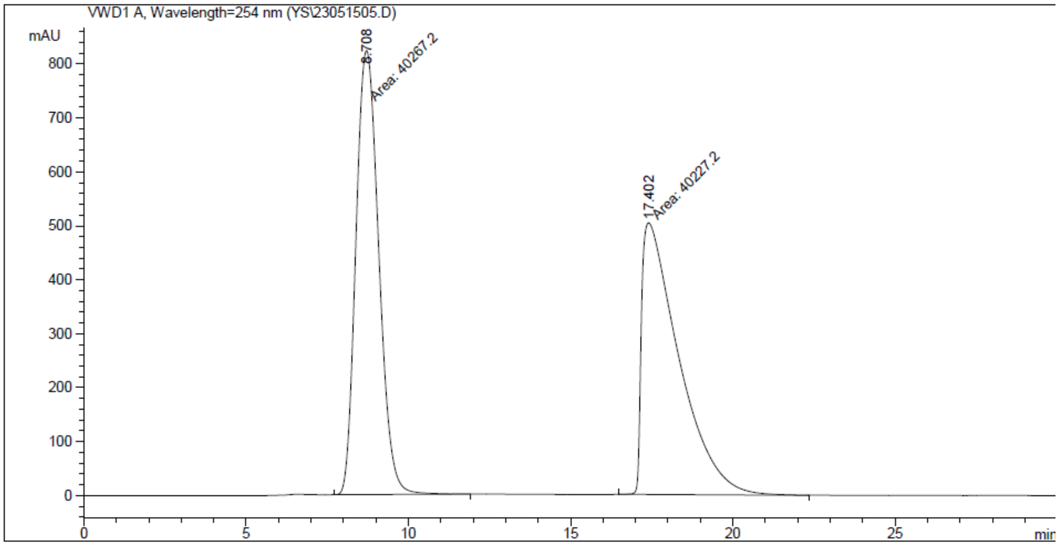

Area Percent Report

Peak #	RetTime [min]	Type	Width [min]	Area mAU *s	Height [mAU]	Area %
1	8.708	MM	0.8148	4.02672e4	823.70349	50.0248
2	17.402	MM	1.3305	4.02272e4	503.91519	49.9752
Totals :				8.04944e4	1327.61868	

enantiomerically enriched

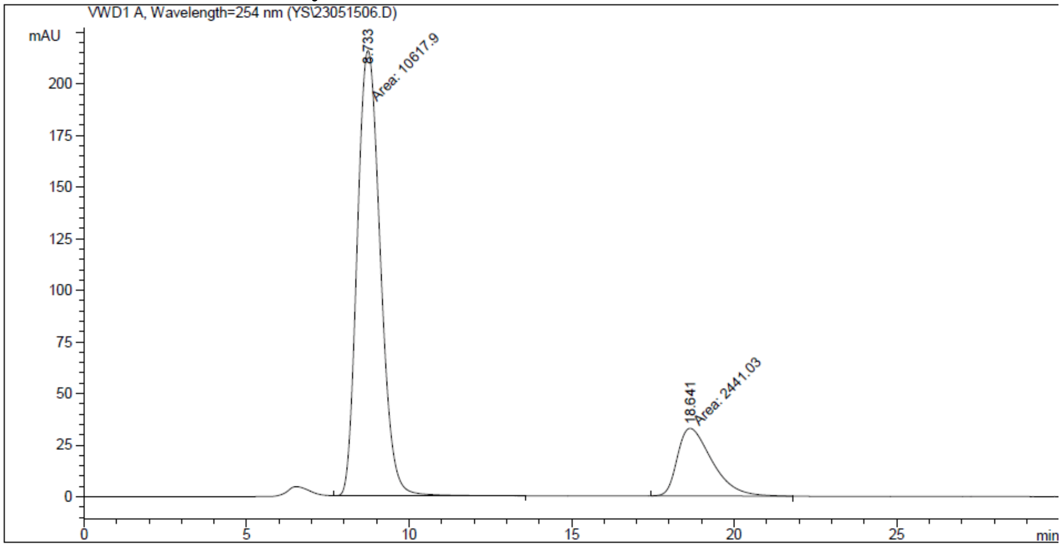

Area Percent Report

Peak #	RetTime [min]	Type	Width [min]	Area mAU *s	Height [mAU]	Area %
1	8.733	MM	0.8202	1.06179e4	215.76010	81.3076
2	18.641	MM	1.2404	2441.02588	32.79787	18.6924
Totals :				1.30589e4	248.55798	

HPLC analysis

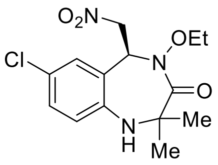

3c

racemic

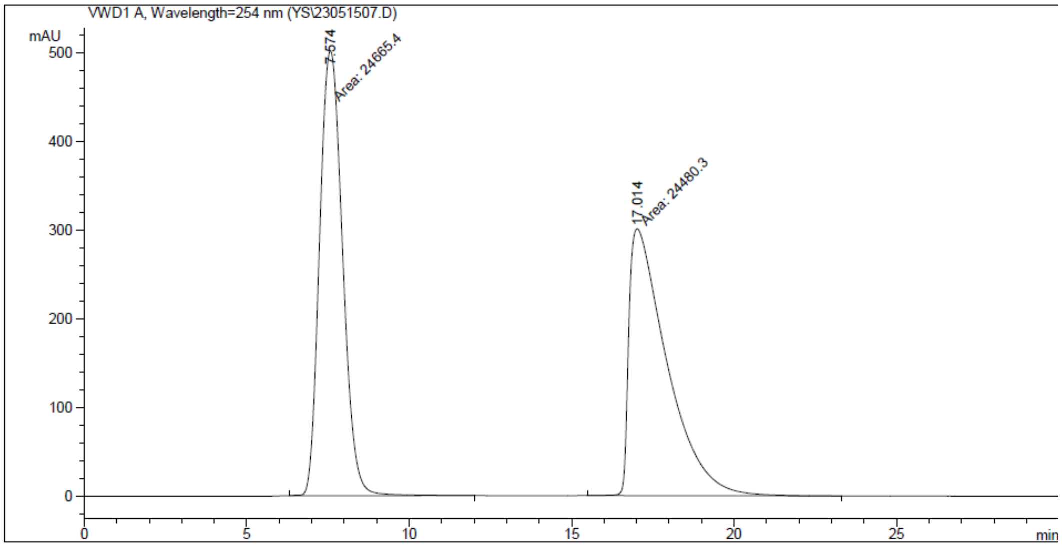

Area Percent Report

Peak #	RetTime [min]	Type	Width [min]	Area mAU *s	Height [mAU]	Area %
1	7.574	MM	0.8193	2.46654e4	501.74557	50.1884
2	17.014	MM	1.3561	2.44803e4	300.87616	49.8116
Totals :				4.91457e4	802.62173	

enantiomerically enriched

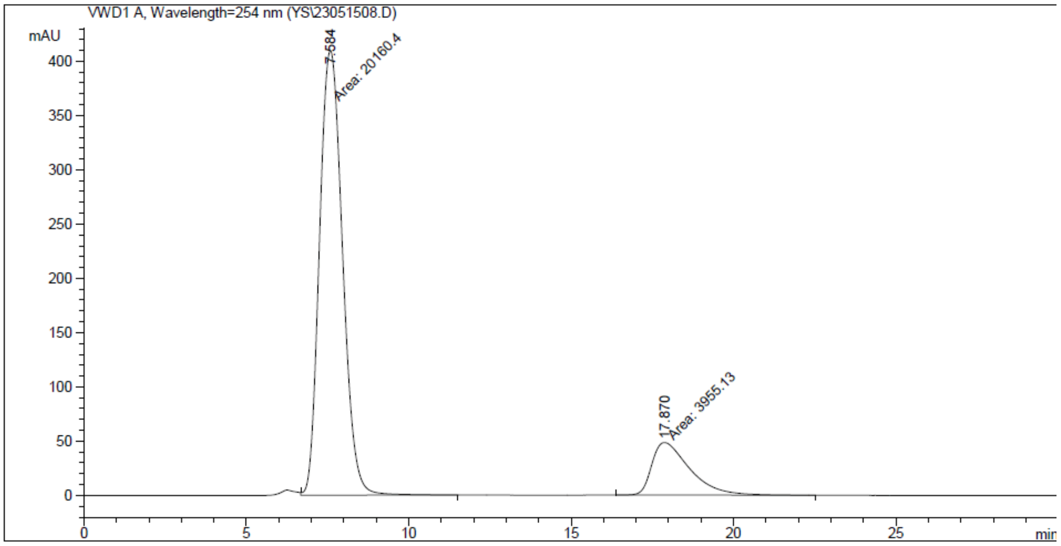

Area Percent Report

Peak #	RetTime [min]	Type	Width [min]	Area mAU *s	Height [mAU]	Area %
1	7.584	FM	0.8196	2.01604e4	409.96609	83.5993
2	17.870	MM	1.3591	3955.12817	48.50093	16.4007
Totals :				2.41155e4	458.46702	

HPLC analysis

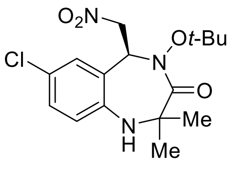

3d

racemic

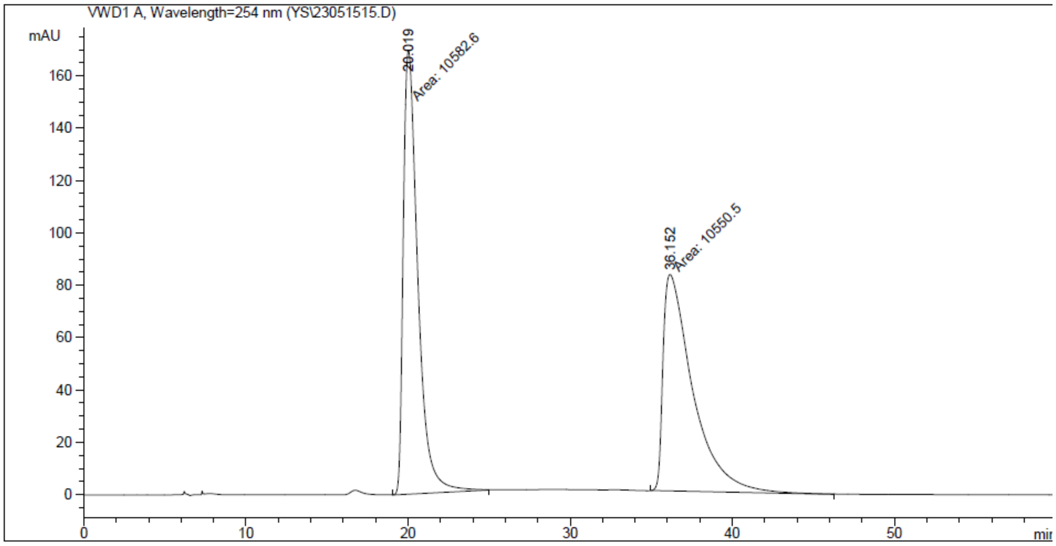


```
=====================================================================
                        Area Percent Report
=====================================================================

Peak RetTime Type  Width     Area       Height     Area
  #   [min]        [min]   mAU    *s   [mAU   ]      %
----|-------|----|-------|----------|----------|--------|
   1  20.019 MM    1.0401 1.05826e4  169.57364 50.0761
   2  36.152 MM    2.1254 1.05505e4   82.73172 49.9239

Totals :                  2.11331e4  252.30536
```

enantiomerically enriched

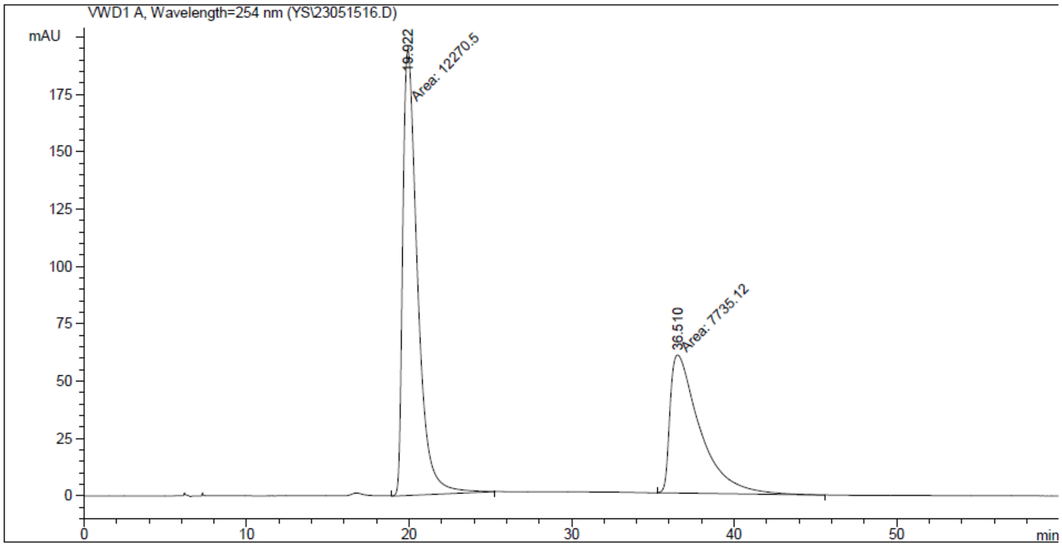


```
=====================================================================
                        Area Percent Report
=====================================================================

Peak RetTime Type  Width     Area       Height     Area
  #   [min]        [min]   mAU    *s   [mAU   ]      %
----|-------|----|-------|----------|----------|--------|
   1  19.922 MM    1.0533 1.22705e4  194.15288 61.3353
   2  36.510 MM    2.1384 7735.12256   60.28795 38.6647

Totals :                  2.00056e4  254.44083
```

HPLC analysis

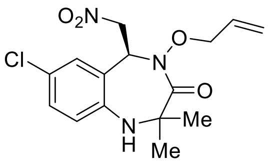

3e

racemic

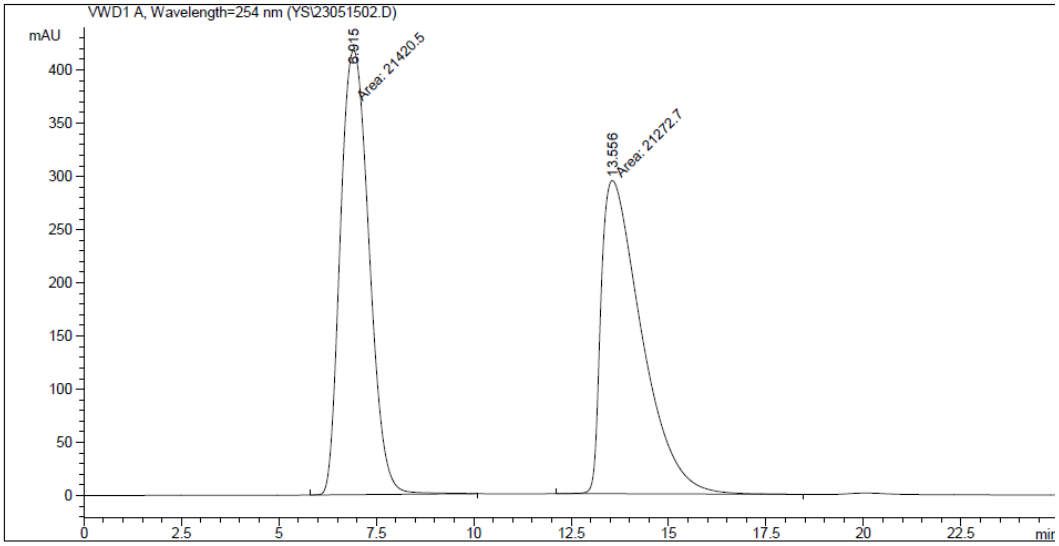

Area Percent Report

Peak #	RetTime [min]	Type	Width [min]	Area mAU *s	Height [mAU]	Area %
1	6.915	MM	0.8540	2.14205e4	418.04431	50.1731
2	13.556	MM	1.2037	2.12727e4	294.53772	49.8269
Totals :				4.26932e4	712.58203	

enantiomerically enriched

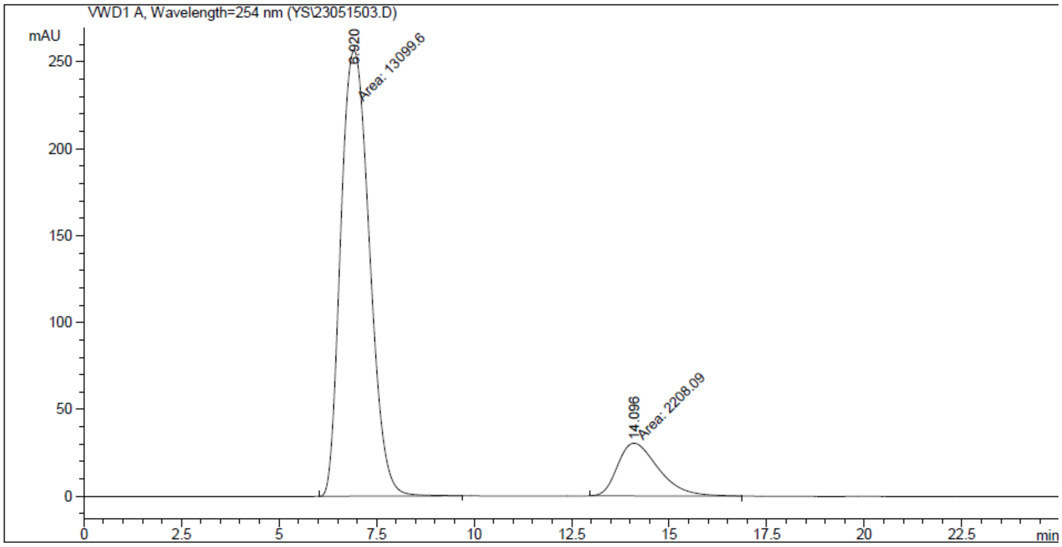

Area Percent Report

Peak #	RetTime [min]	Type	Width [min]	Area mAU *s	Height [mAU]	Area %
1	6.920	MM	0.8512	1.30996e4	256.47943	85.5752
2	14.096	MM	1.2125	2208.09473	30.35223	14.4248
Totals :				1.53077e4	286.83166	

HPLC analysis

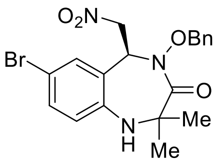

3f

racemic

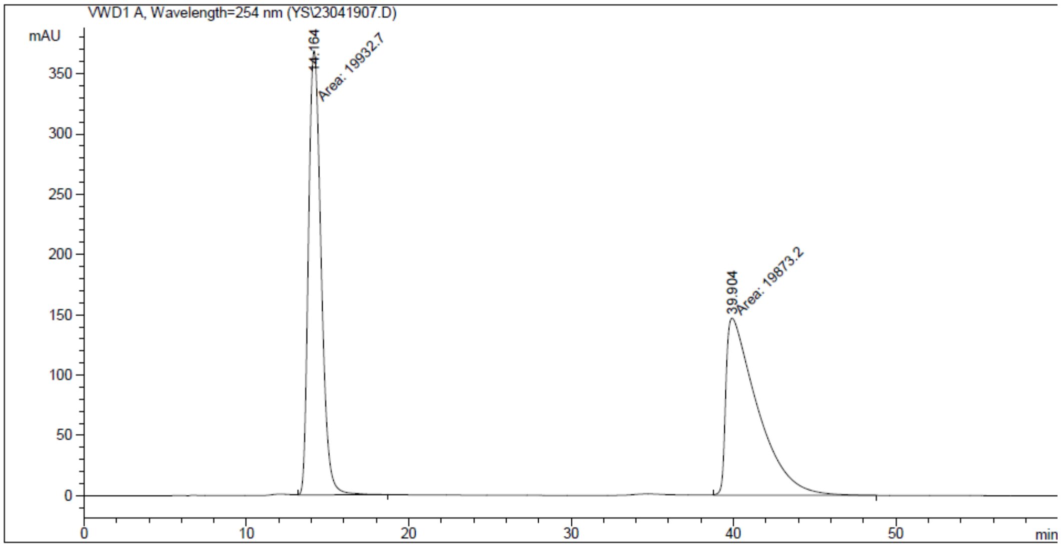

Area Percent Report

Peak #	RetTime [min]	Type	Width [min]	Area mAU *s	Height [mAU]	Area %
1	14.164	MM	0.9009	1.99327e4	368.76813	50.0748
2	39.904	MM	2.2546	1.98732e4	146.90877	49.9252
Totals :				3.98059e4	515.67690	

enantiomerically enriched

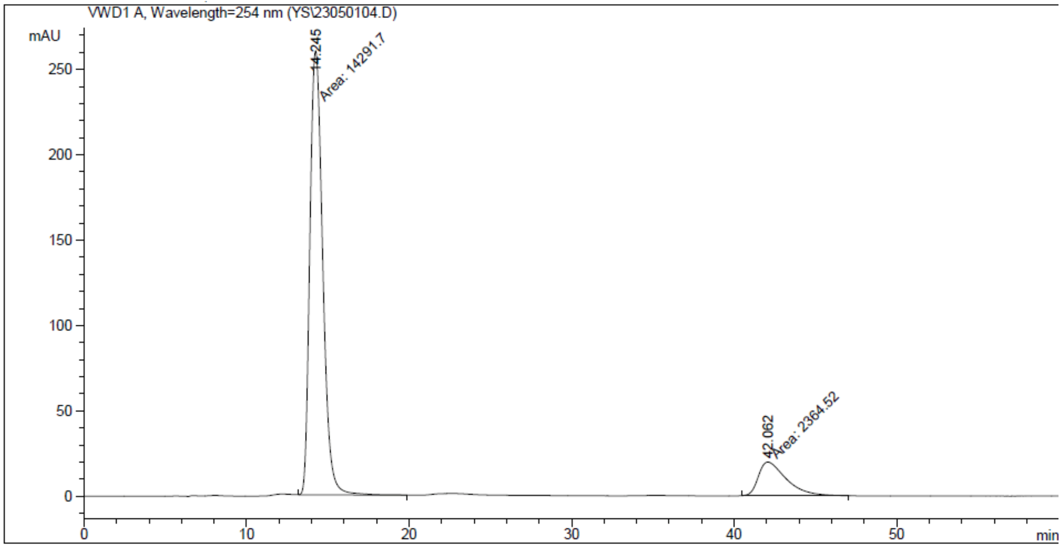

Area Percent Report

Peak #	RetTime [min]	Type	Width [min]	Area mAU *s	Height [mAU]	Area %
1	14.245	MM	0.9150	1.42917e4	260.30850	85.8040
2	42.062	MM	1.9949	2364.52319	19.75484	14.1960
Totals :				1.66562e4	280.06334	

HPLC analysis

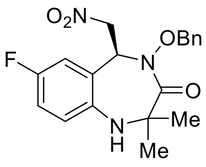

3g

racemic

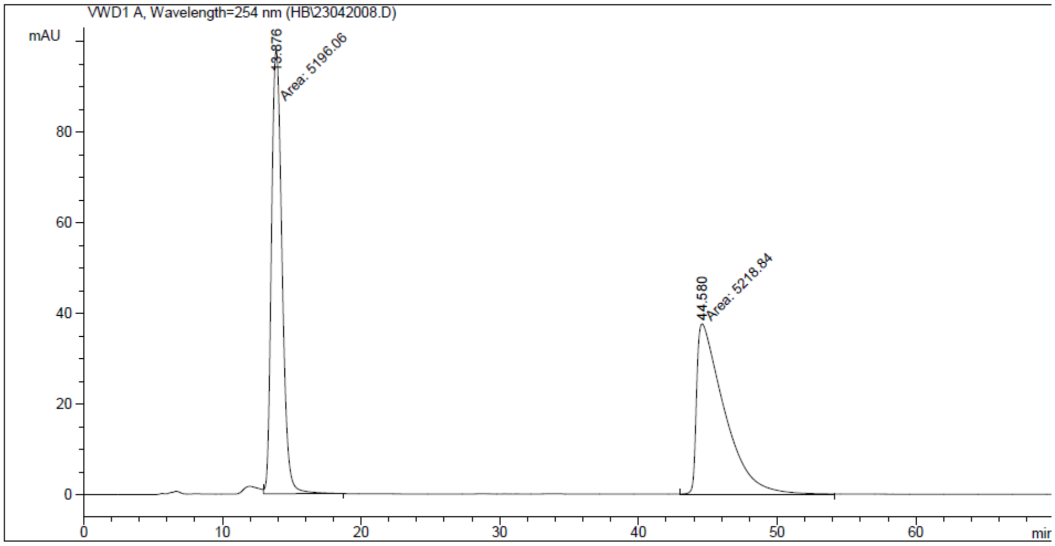

Area Percent Report

Peak #	RetTime [min]	Type	Width [min]	Area mAU *s	Height [mAU]	Area %
1	13.876	FM	0.8845	5196.06250	97.90967	49.8906
2	44.580	MM	2.3117	5218.84375	37.62687	50.1094
Totals :				1.04149e4	135.53653	

enantiomerically enriched

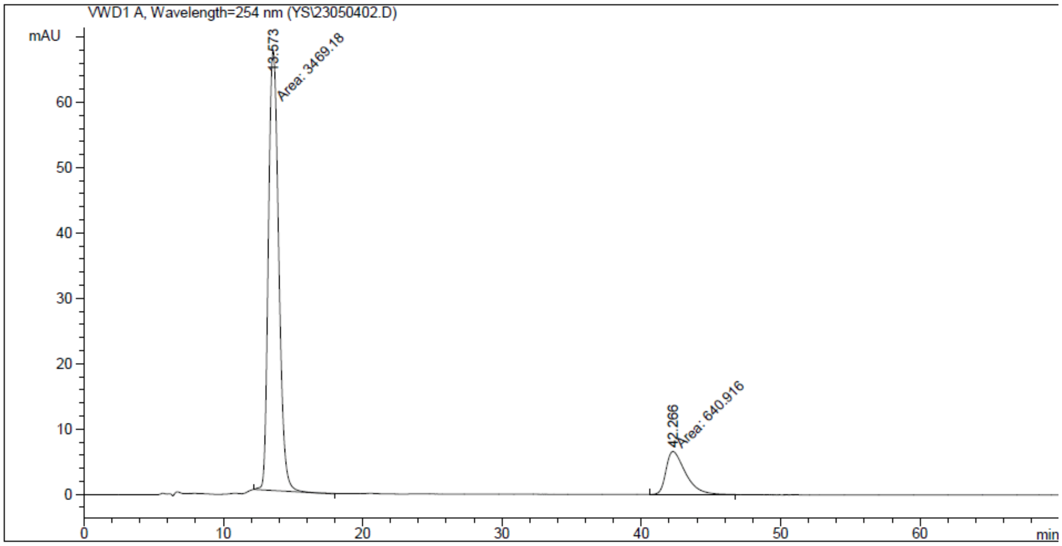

Area Percent Report

Peak #	RetTime [min]	Type	Width [min]	Area mAU *s	Height [mAU]	Area %
1	13.573	MM	0.8582	3469.18042	67.37269	84.4063
2	42.266	MM	1.6223	640.91589	6.58448	15.5937
Totals :				4110.09631	73.95716	

HPLC analysis

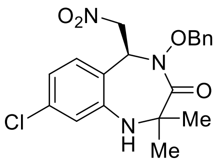

3h

racemic

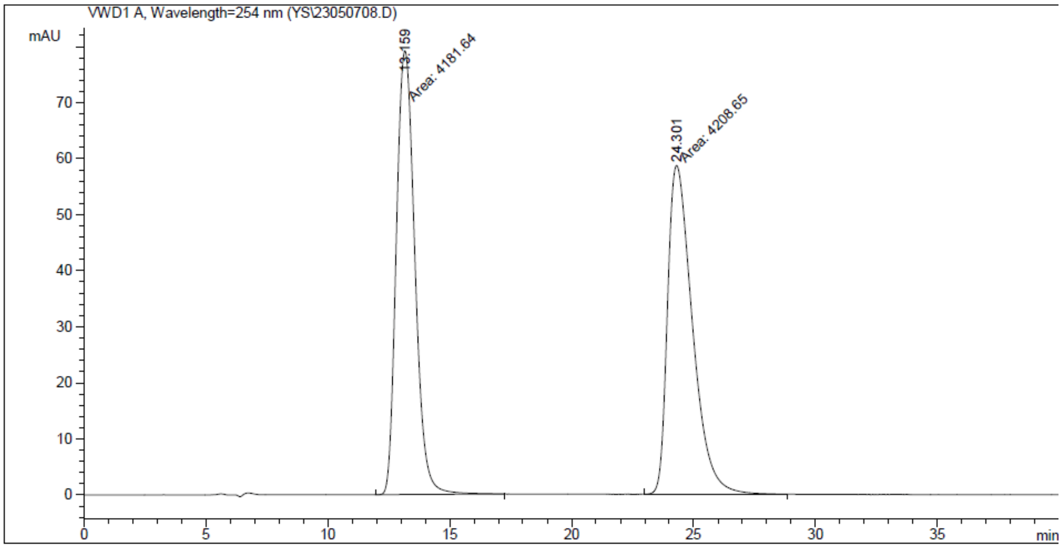

Area Percent Report

Peak #	RetTime [min]	Type	Width [min]	Area mAU *s	Height [mAU]	Area %
1	13.159	MM	0.8797	4181.63721	79.22694	49.8390
2	24.301	MM	1.1926	4208.65234	58.81641	50.1610
Totals :				8390.28955	138.04336	

enantiomerically enriched

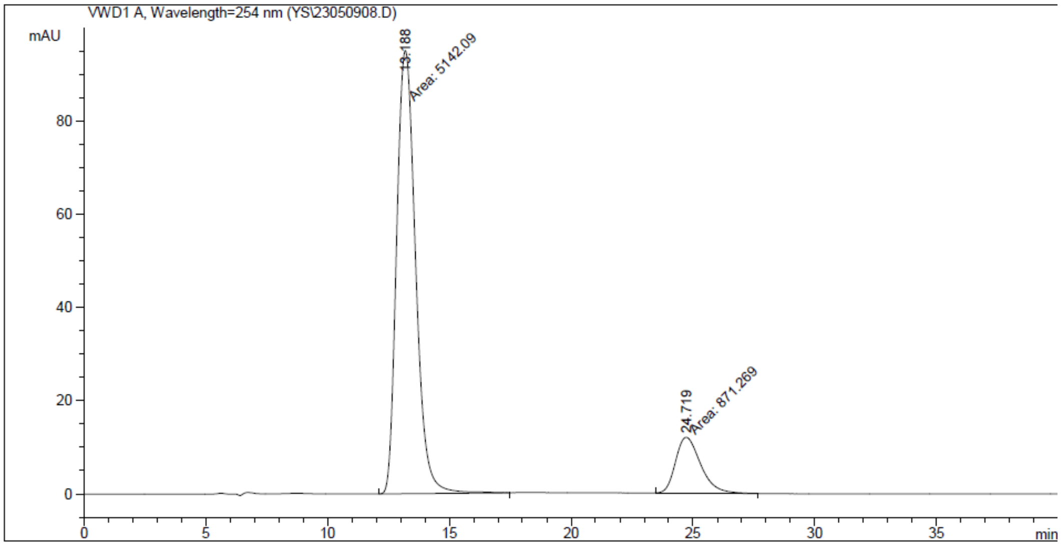

Area Percent Report

Peak #	RetTime [min]	Type	Width [min]	Area mAU *s	Height [mAU]	Area %
1	13.188	MM	0.9002	5142.08936	95.19852	85.5111
2	24.719	MM	1.2056	871.26929	12.04434	14.4889
Totals :				6013.35864	107.24286	

HPLC analysis

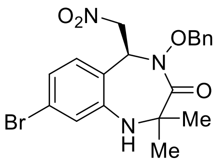

3i

racemic

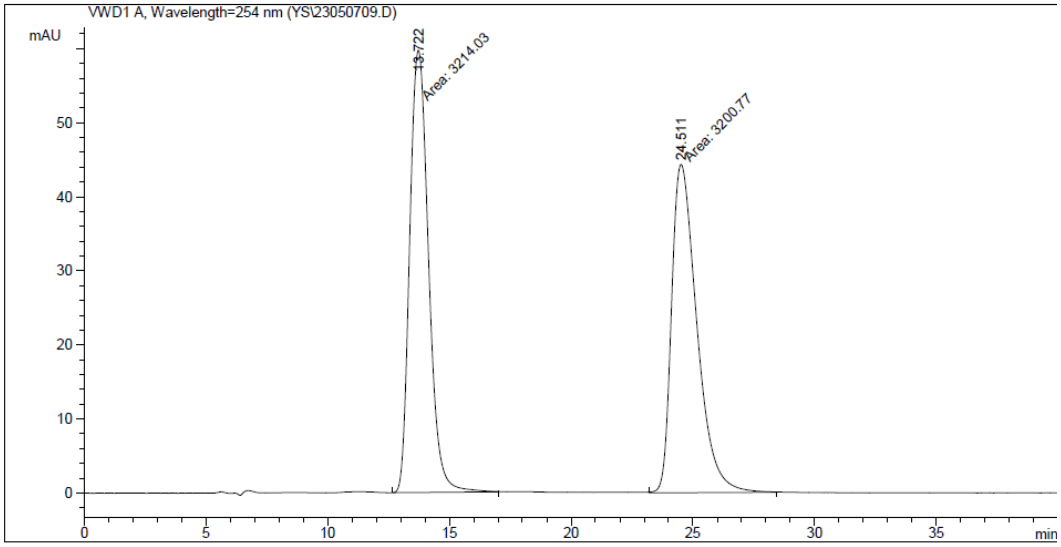

Area Percent Report

Peak #	RetTime [min]	Type	Width [min]	Area mAU *s	Height [mAU]	Area %
1	13.722	MM	0.8964	3214.03198	59.76060	50.1034
2	24.511	MM	1.2030	3200.77124	44.34533	49.8966
Totals :				6414.80322	104.10593	

enantiomerically enriched

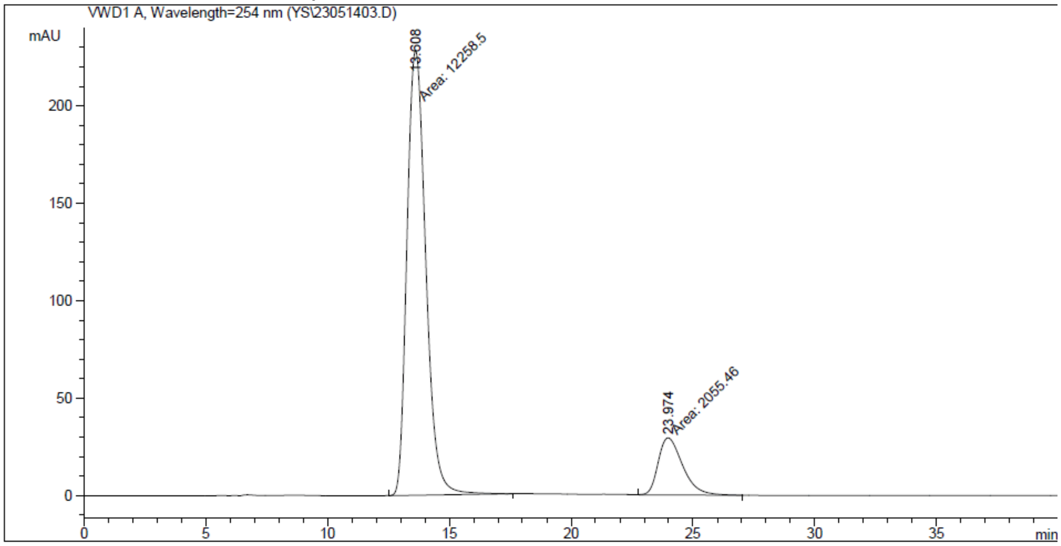

Area Percent Report

Peak #	RetTime [min]	Type	Width [min]	Area mAU *s	Height [mAU]	Area %
1	13.608	MM	0.8948	1.22585e4	228.34015	85.6402
2	23.974	MM	1.1683	2055.45728	29.32274	14.3598
Totals :				1.43140e4	257.66289	

HPLC analysis

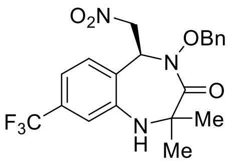

3j

racemic

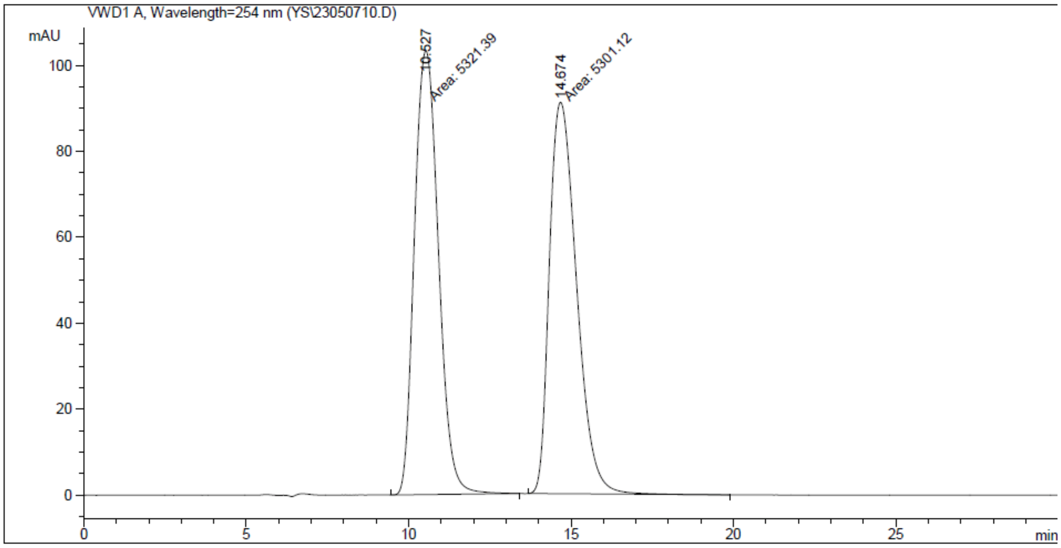

Area Percent Report

Peak #	RetTime [min]	Type	Width [min]	Area mAU *s	Height [mAU]	Area %
1	10.527	MM	0.8574	5321.39014	103.43745	50.0954
2	14.674	MM	0.9687	5301.11768	91.20477	49.9046
Totals :				1.06225e4	194.64223	

enantiomerically enriched

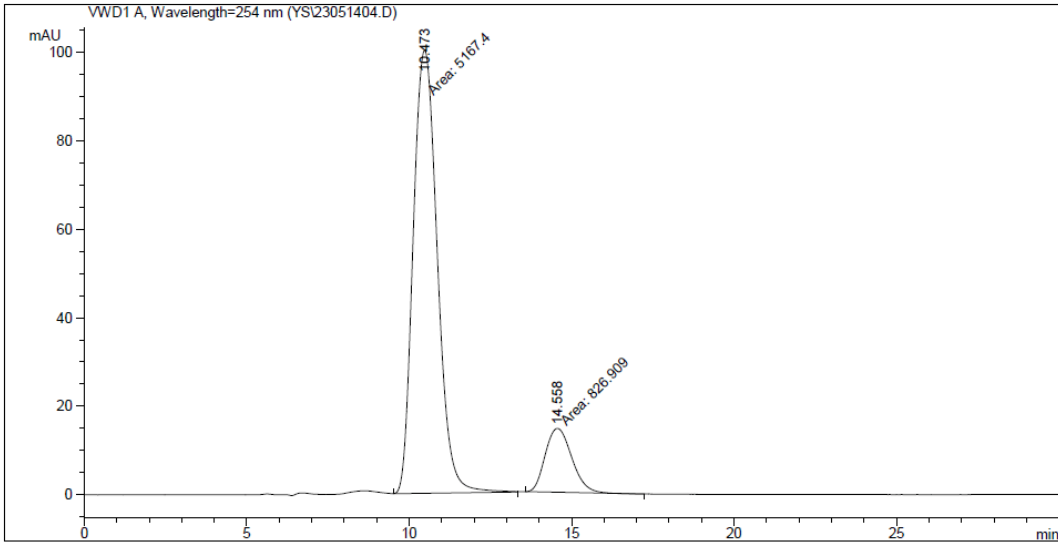

Area Percent Report

Peak #	RetTime [min]	Type	Width [min]	Area mAU *s	Height [mAU]	Area %
1	10.473	MM	0.8587	5167.40332	100.29713	86.2051
2	14.558	MM	0.9522	826.90869	14.47383	13.7949
Totals :				5994.31201	114.77097	

HPLC analysis

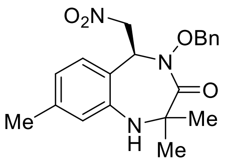

3k

racemic

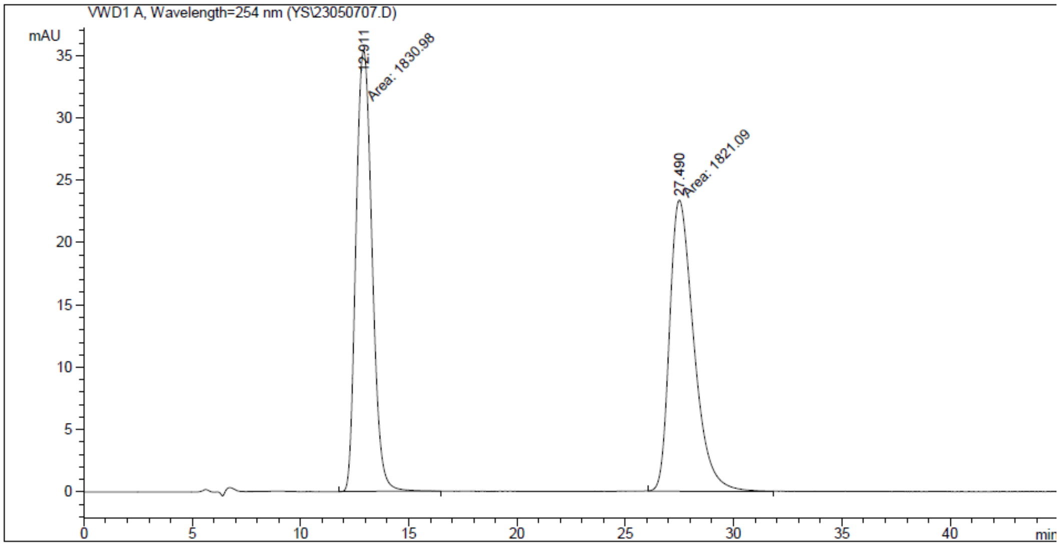

Area Percent Report

Peak #	RetTime [min]	Type	Width [min]	Area mAU *s	Height [mAU]	Area %
1	12.911	MM	0.8620	1830.98108	35.40203	50.1354
2	27.490	MM	1.2986	1821.09131	23.37189	49.8646
Totals :				3652.07239	58.77391	

enantiomerically enriched

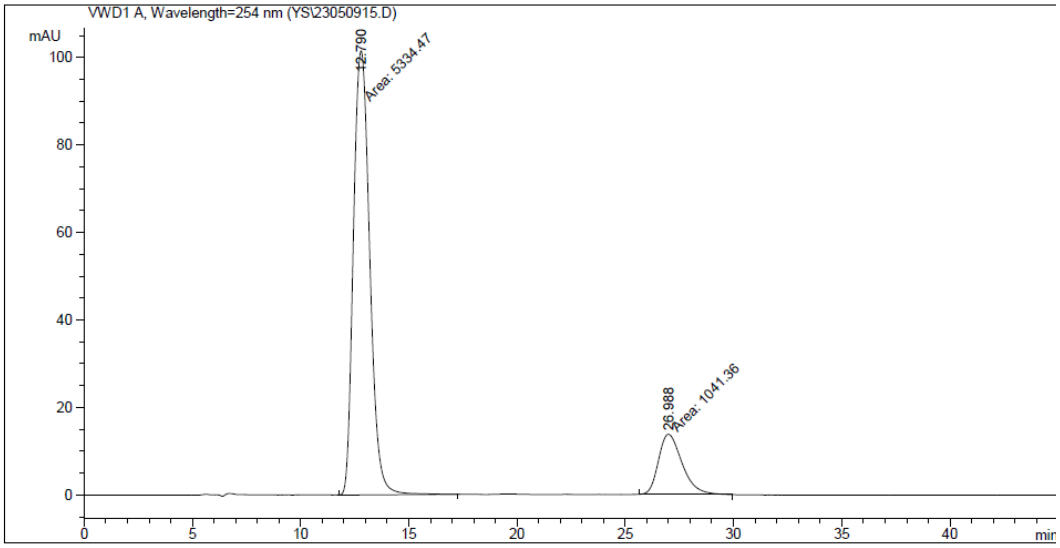

Area Percent Report

Peak #	RetTime [min]	Type	Width [min]	Area mAU *s	Height [mAU]	Area %
1	12.790	MM	0.8751	5334.46680	101.59209	83.6671
2	26.988	MM	1.2611	1041.36035	13.76215	16.3329
Totals :				6375.82715	115.35424	

HPLC analysis

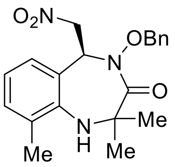

3l

racemic

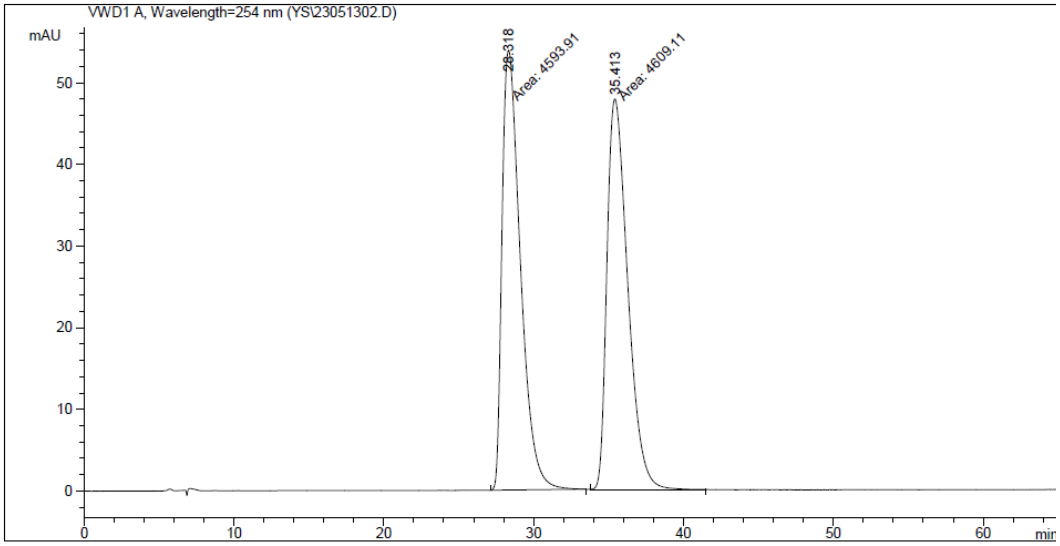


```
=====================================================================
                         Area Percent Report
=====================================================================
```

Peak #	RetTime [min]	Type	Width [min]	Area mAU *s	Height [mAU]	Area %
1	28.318	MM	1.4200	4593.91211	53.91792	49.9174
2	35.413	MM	1.6048	4609.10645	47.86654	50.0826
Totals :				9203.01855	101.78445	

enantiomerically enriched

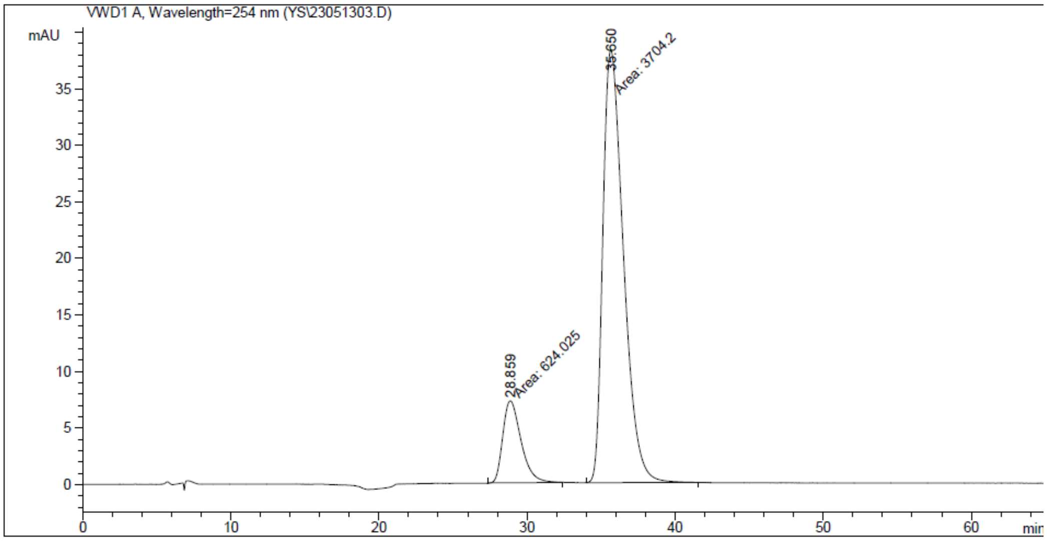


```
=====================================================================
                         Area Percent Report
=====================================================================
```

Peak #	RetTime [min]	Type	Width [min]	Area mAU *s	Height [mAU]	Area %
1	28.859	MM	1.4325	624.02466	7.26046	14.4176
2	35.650	MM	1.6128	3704.19897	38.28032	85.5824
Totals :				4328.22363	45.54078	

HPLC analysis

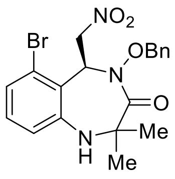

3m

racemic

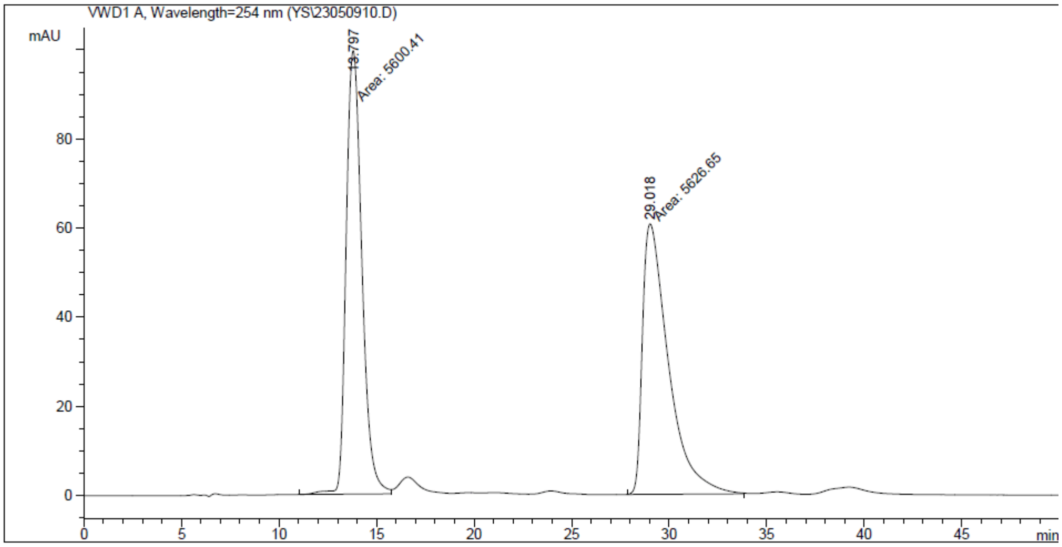

Area Percent Report

Peak #	RetTime [min]	Type	Width [min]	Area mAU *s	Height [mAU]	Area %
1	13.797	MF	0.9376	5600.41357	99.54832	49.8832
2	29.018	MM	1.5453	5626.64844	60.68752	50.1168

Totals : 1.12271e4 160.23584

enantiomerically enriched

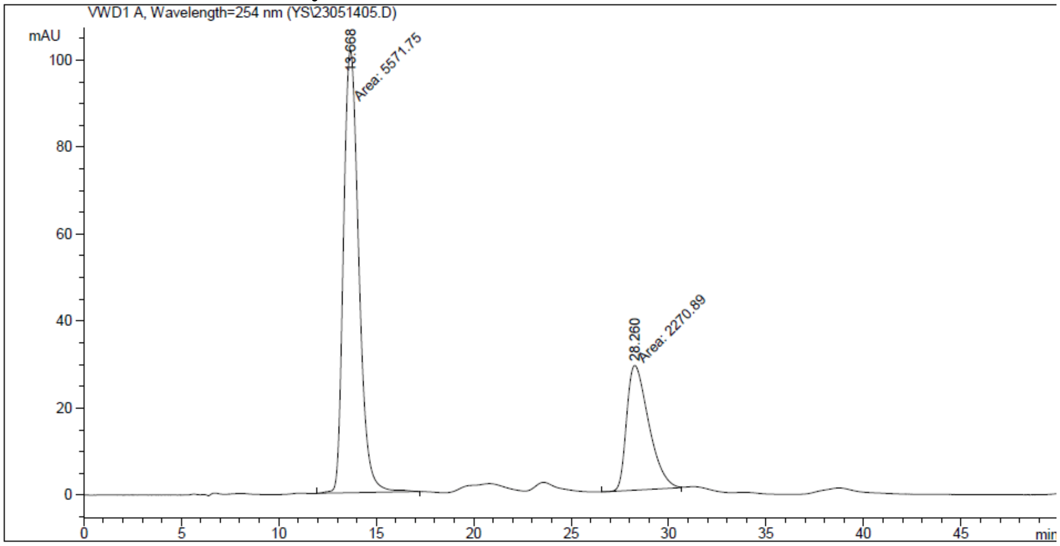

Area Percent Report

Peak #	RetTime [min]	Type	Width [min]	Area mAU *s	Height [mAU]	Area %
1	13.668	MM	0.9123	5571.74756	101.78458	71.0443
2	28.260	MM	1.3176	2270.88574	28.72473	28.9557

Totals : 7842.63330 130.50931

HPLC analysis

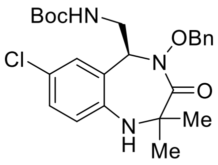

4

racemic

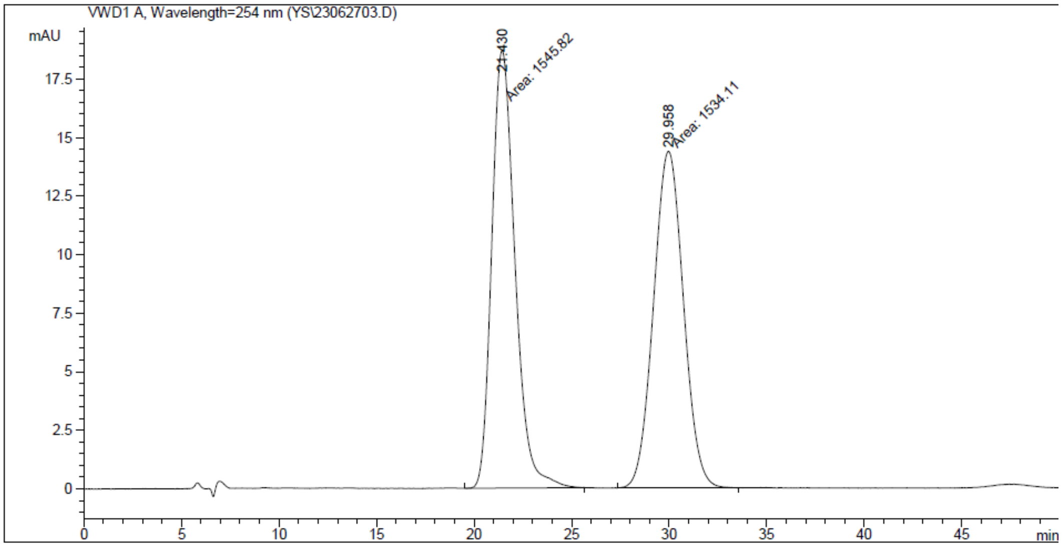


```
=====================================================================
                        Area Percent Report
=====================================================================

Peak RetTime Type  Width     Area      Height     Area
  #   [min]        [min]  mAU    *s  [mAU    ]     %
----|-------|----|-------|----------|----------|--------|
   1  21.430 MM    1.3792 1545.82251   18.67967  50.1901
   2  29.958 MM    1.7782 1534.11182   14.37905  49.8099

Totals :                  3079.93433   33.05872
```

enantiomerically enriched

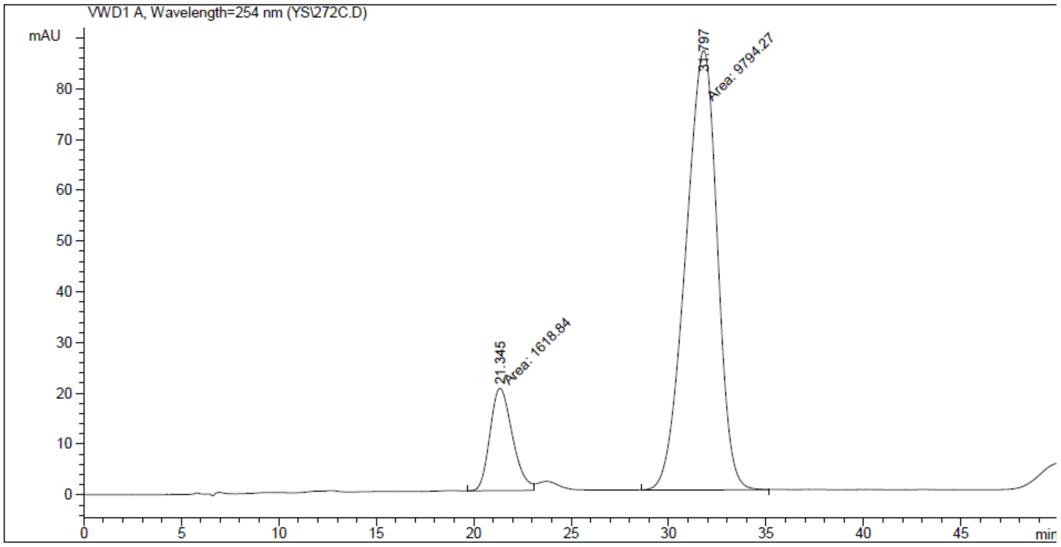


```
=====================================================================
                        Area Percent Report
=====================================================================

Peak RetTime Type  Width     Area      Height     Area
  #   [min]        [min]  mAU    *s  [mAU    ]     %
----|-------|----|-------|----------|----------|--------|
   1  21.345 MF    1.3360 1618.83679   20.19582  14.1840
   2  31.797 MM    1.8847 9794.26855   86.61343  85.8160

Totals :                  1.14131e4   106.80925
```

HPLC analysis

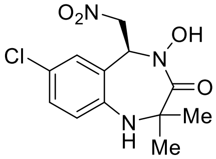

5

racemic

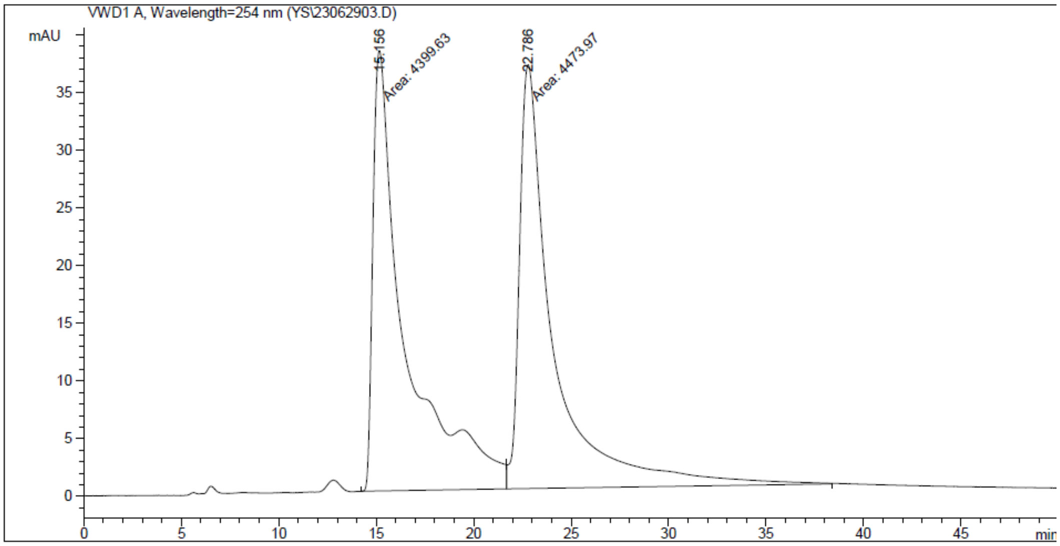

Area Percent Report

Peak #	RetTime [min]	Type	Width [min]	Area mAU *s	Height [mAU]	Area %
1	15.156	MF	1.9192	4399.63086	38.20783	49.5811
2	22.786	FM	2.0278	4473.97461	36.77242	50.4189
Totals :				8873.60547	74.98025	

enantiomerically enriched

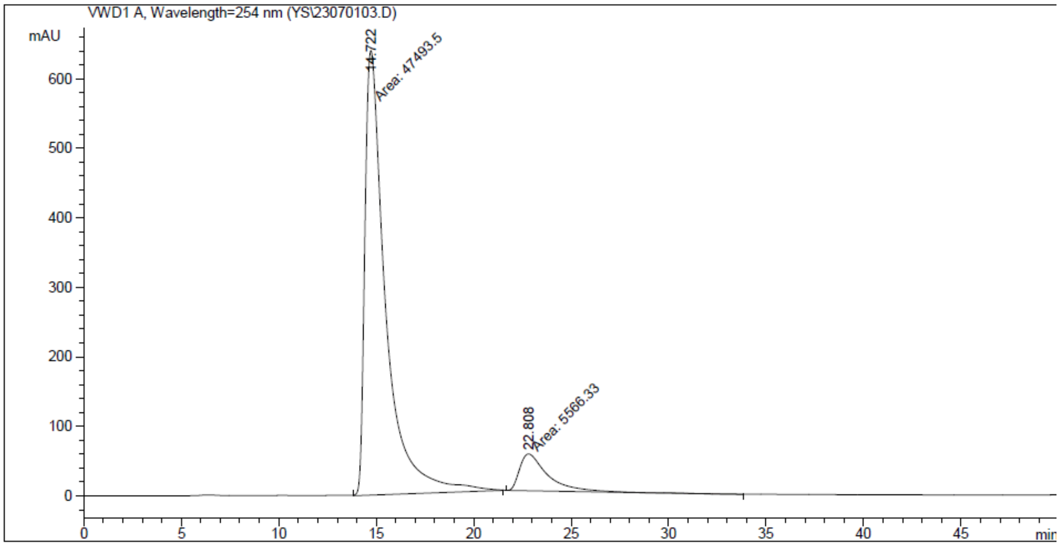

Area Percent Report

Peak #	RetTime [min]	Type	Width [min]	Area mAU *s	Height [mAU]	Area %
1	14.722	MM	1.2365	4.74935e4	640.15240	89.5093
2	22.808	MM	1.7447	5566.32813	53.17291	10.4907
Totals :				5.30599e4	693.32532	
